# Ruthenium-cobalt single atom alloy for CO photo-hydrogenation to liquid fuels at ambient pressures

**DOI:** 10.1038/s41467-023-37631-5

**Published:** 2023-04-05

**Authors:** Jiaqi Zhao, Jinjia Liu, Zhenhua Li, Kaiwen Wang, Run Shi, Pu Wang, Qing Wang, Geoffrey I. N. Waterhouse, Xiaodong Wen, Tierui Zhang

**Affiliations:** 1 Key Laboratory of Photochemical Conversion and Optoelectronic Materials, Technical Institute of Physics and Chemistry, Chinese Academy of Sciences, Beijing, 100190 China; 2 Center of Materials Science and Optoelectronics Engineering, University of Chinese Academy of Sciences, Beijing, 100049 China; 3 State Key Laboratory of Coal Conversion, Institute of Coal Chemistry, Chinese Academy of Sciences, Taiyuan, 030001 China; 4 National Energy Center for Coal to Clean Fuels, Synfuels China Co., Ltd, Beijing, 101400 China; 5 Beijing Key Lab of Microstructure and Properties of Advanced Materials, Beijing University of Technology, Beijing, 100124 China; 6 School of Chemical Sciences, The University of Auckland, Auckland, 1142 New Zealand

**Keywords:** Photocatalysis, Nanoparticles, Heterogeneous catalysis

## Abstract

Photothermal Fischer-Tropsch synthesis represents a promising strategy for converting carbon monoxide into value-added chemicals. High pressures (2-5 MPa) are typically required for efficient C-C coupling reactions and the production of C_5+_ liquid fuels. Herein, we report a ruthenium-cobalt single atom alloy (Ru_1_Co-SAA) catalyst derived from a layered-double-hydroxide nanosheet precursor. Under UV-Vis irradiation (1.80 W cm^−2^), Ru_1_Co-SAA heats to 200 °C and photo-hydrogenates CO to C_5+_ liquid fuels at ambient pressures (0.1-0.5 MPa). Single atom Ru sites dramatically enhance the dissociative adsorption of CO, whilst promoting C-C coupling reactions and suppressing over-hydrogenation of CH_x_* intermediates, resulting in a CO photo-hydrogenation turnover frequency of 0.114 s^−1^ with 75.8% C_5+_ selectivity. Owing to the local Ru-Co coordination, highly unsaturated intermediates are generated during C-C coupling reactions, thereby improving the probability of carbon chain growth into C_5+_ liquid fuels. The findings open new vistas towards C_5+_ liquid fuels under sunlight at mild pressures.

## Abstract (web summary)

Photothermal Fischer-Tropsch synthesis represents a promising strategy for converting CO into value-added chemicals. Here the authors report a Ru_1_Co single atom alloy catalyst for CO photo-hydrogenation to C_5+_ liquid fuels at ambient pressure, and confirm the role of Ru single atoms in promoting CO activation and C-C coupling whilst suppressing CH_x_* over-hydrogenation.

## Introduction

Efficient utilization and capture of solar energy is critical to meeting the energy needs of future societies^1^. Recently, photo-driven Fischer–Tropsch synthesis (FTS) reactions have attracted a lot of attention as an energy-efficient route towards value-added chemicals^2,3^. A number of reported works have demonstrated that efficient photothermal CO hydrogenation to alkanes and alkenes is possible using UV-Vis lamps or concentrated direct sunlight as the heating source^4–8^. To date, most of these works have only yielded light hydrocarbons (typically C_1_-C_5_ alkanes or C_2_-C_4_ alkenes). A prized yet challenging target of FTS research is the synthesis of value-added C_5+_ hydrocarbon liquid fuels. Further, both thermal and photothermal FTS demand relatively harsh conditions, typically high pressures (2-5 MPa)^9–18^. Economic incentives therefore exist to discover photo-driven CO hydrogenation catalysts that deliver C_5+_ hydrocarbon liquid fuels at near ambient pressures (below 0.5 MPa).

FTS reactions are complex multi-step processes, involving CO adsorption and hydrogenation to CH_x_* intermediates followed by C-C coupling and further hydrogenation steps. Product selectivity is largely controlled by the probability of C-C coupling reactions relative to over-hydrogenation reactions. Supported Co, Ru, Fe and Ni nanoparticles are commonly used as metal catalysts in FTS, each of which differs in its electronic structure, CO hydrogenation activity, FTS product selectivity and stability. Co-based and Ru-based catalysts show good activity for C-C coupling reactions at high pressures, and therefore are used in conventional thermal FTS to produce liquid fuels (including gasoline: C_5-12_ hydrocarbons, jet fuel: C_8-16_ hydrocarbons, and diesel: C_13-20_ hydrocarbons)^19–21^. However, if FTS reactions are carried out at low pressures using the same catalysts, the chain growth probability is significant reduced, resulting in the poor C_5+_ selectivity^22^. Recently, combining Co-based catalysts with Ru, Pt, Pd, and Rh to create bimetallic catalysts has proved effective in promoting CO conversion whilst hardly enhancing C_5+_ selectivity^14,15,23–25^. However, alloy composition and element distribution in alloy nanoparticles are critical. The low energy barrier of H_2_ dissociation on Ru-rich domains can lead to the over-hydrogenation of CH_x_* intermediates, resulting in CH_4_ production rather than C-C coupling reactions at near ambient pressures^26–30^. The low intrinsic C-C coupling ability of traditional Co-based, Ru-based and RuCo-alloyed catalysts necessitates operation at high pressures to increase the coupling probability. Therefore, novel Co-Ru alloy designs which precisely regulate the balance between C-C coupling and methanation processes are essential for achieving a high selectivity towards C_5+_ liquid fuels during CO photo-hydrogenation at near ambient pressures.

Single atom alloys (SAAs), a novel bimetallic subset of metal single atom catalysts (SACs), contain isolated metal atoms of one metal dispersed in the surface of another metal (the host)^31,32^. SAAs offer unique catalytic properties compared to single metal catalysts or conventional binary alloy catalysts in selective hydrogenation and C-C coupling reactions, including improved activity, selectivity, and resistance to deactivation via coke formation^33,34^. Layered-double-hydroxide (LDH) nanosheets due to their 2D structure and compositional flexibility are now widely used in the fabrication of novel SACs for the energy sector^35–38^. We hypothesized that the topological structural transformation of LDH nanosheets with heating in the hydrogen atmosphere should allow the rational design of alumina-supported SAA catalysts (e.g. Co nanoparticles with surface atomically dispersed Ru atoms). The Ru_1_ coordination should lessen the strong hydrogenation ability of Ru-rich domains at low pressures (<0.5 MPa), thus allowing CO photo-hydrogenation to C_5+_ hydrocarbons under extremely mild conditions.

Herein, we report the successful fabrication of an alumina-supported RuCo single atom alloy catalyst (Ru_1_Co-SAA) with atomically dispersed Ru sites in face-centered cubic (fcc) Co nanoparticles. Ru_1_Co-SAA is synthesized by hydrogen reduction of Ru_1_CoAl-LDH nanosheets at 650 °C, and demonstrates outstanding performance for the CO photo-hydrogenation to C_5+_ liquid fuels under ultraviolet-visible (UV-Vis) irradiation. Isolated Ru atoms on the surface of the metallic Co nanoparticles are confirmed by the transmission electron microscopy (TEM) and Ru K-edge X-ray absorption spectroscopy (XAS) characterization studies. Under UV-Vis light irradiation (1.80 W cm^−2^) and near ambient pressures (0.5 MPa), Ru_1_Co-SAA offers a CO conversion of 58.6% (CO turnover frequency, TOF, of 0.114 s^−1^) and a remarkable C_5+_ selectivity of 75.8%, significantly outperforming catalysts based on alumina-supported Co nanoparticles (Co-NP) or RuCo nanoalloys (Ru_n_Co-NA). Impressively, the performance of Ru_1_Co-SAA at low pressures is comparable to state-of-the-art Co-based, Ru-based and RuCo-alloyed FTS catalysts that operate at much higher pressures (normally above 2 MPa). Chemisorption experiments and density functional theory (DFT) calculations reveal that the unique Ru-Co coordination in Ru_1_Co-SAA promotes CO activation and the C-C coupling reactions of CH_x_* intermediates, thus enabling efficient CO photo-hydrogenation activity and C_5+_ selectivity. Moreover, the formation of C_2_* intermediates with high unsaturation over Ru_1_Co-SAA is conducive to C-C coupling to long-chain hydrocarbons (compared to reference catalysts containing Co-NP or Ru_n_Co-NA). To the best of our knowledge, this is the first study to report CO photo-hydrogenation to C_5+_ liquid fuels with high selectivity at ambient and near ambient pressures (0.1-0.5 MPa).

## Results

### Synthesis and characterization of the Ru_1_Co-SAA catalyst

**Fig. 1:**
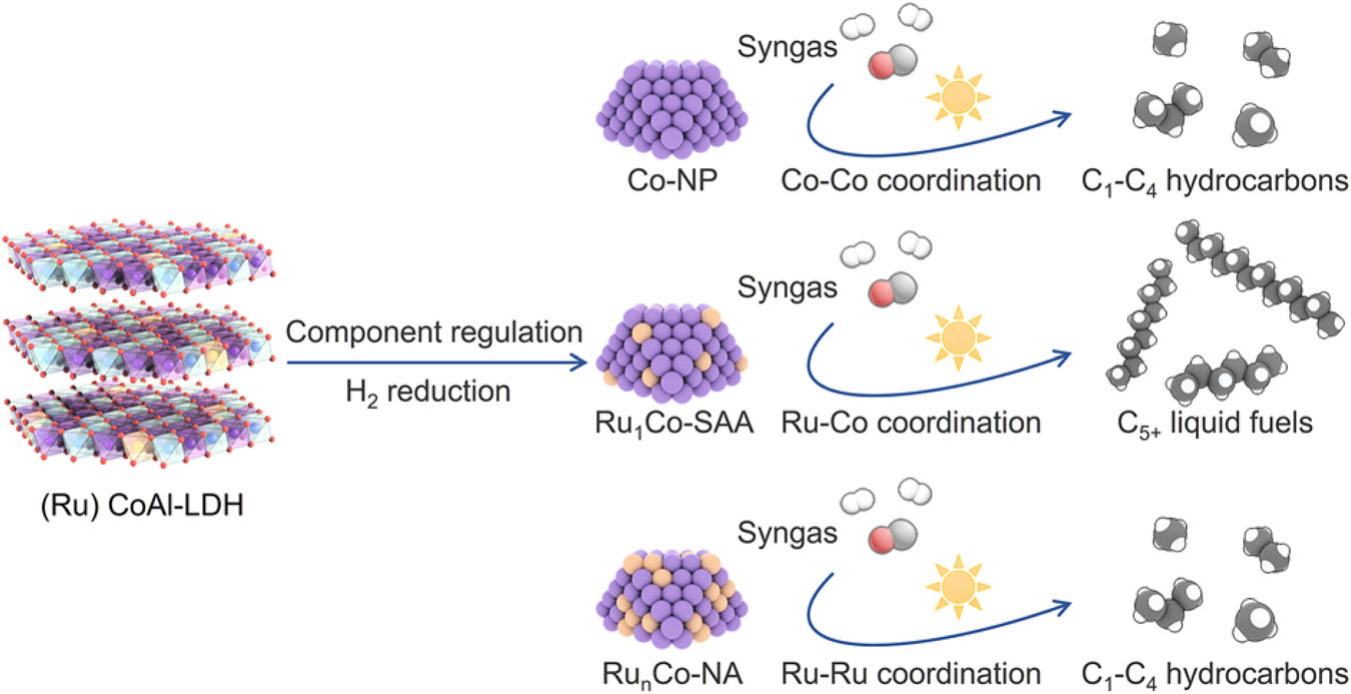
Schematic illustration of the fabrication of the catalysts and the main products of CO photo-hydrogenation. The left illustration shows the structure of LDH precursors. The middle illustration shows nanostructures of active nanoparticles in Co-NP, Ru_1_Co-SAA and Ru_n_Co-NA catalysts derived from LDHs. The right illustration shows that Ru_1_Co-SAA contributes to C_5+_ liquid fuels owing to the dominant Ru-Co coordination while Co-NP and Ru_n_Co-NA lead to C_1_-C_4_ hydrocarbons due to the pure Co-Co coordination and partial Ru-Ru coordination, respectively.

To prepare Ru_1_Co-SAA and Ru_n_Co-NA (Fig. 1), Ru_1_CoAl- and Ru_n_CoAl-LDH nanosheets were firstly synthesized as catalyst precursors using the same one-pot hydrothermal method as CoAl-LDH reported in our previous work^8^. The Ru/Co molar ratios in the precursors and catalysts were measured by inductively coupled plasma-optical emission spectroscopy (ICP-OES, Supplementary Table 1). Powder X-ray diffraction (XRD) results showed that the crystallinity of LDH precursors decreased with increasing Ru loading (Supplementary Fig. 1). Hydrogen temperature-programmed reduction (H_2_-TPR, Supplementary Fig. 2) profiles guided the selection of temperature to reduce the LDH precursors to obtain the Co-NP, Ru_1_Co-SAA and Ru_n_Co-NA catalysts. To ensure that all the cobalt in the precursors was reduced to metallic Co nanoparticles, the LDH precursors were then heated at 650 °C for 5 h in a H_2_/N_2_ (10/90) atmosphere.

**Fig. 2:**
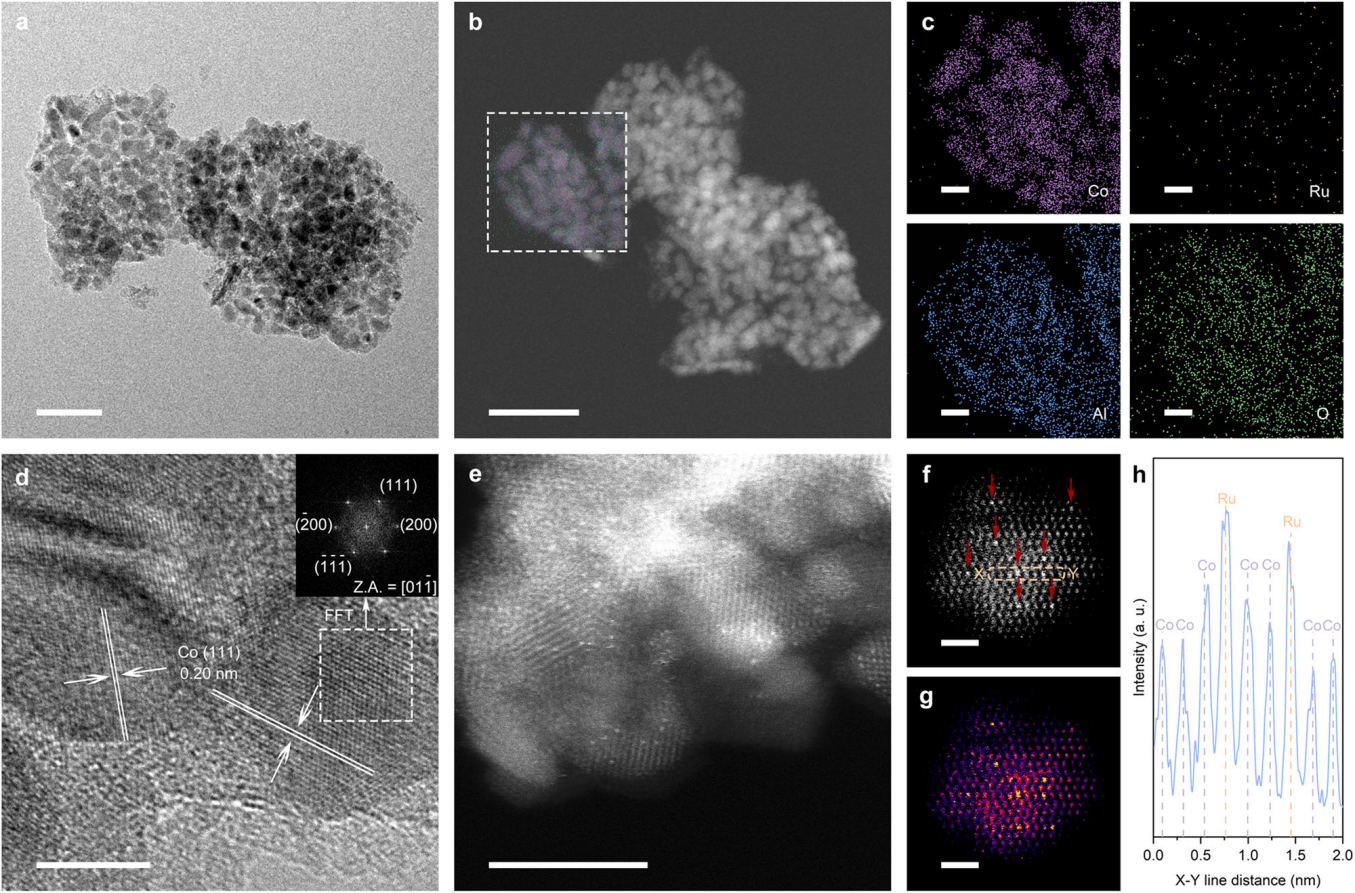
Morphology of the Ru_1_Co-SAA catalyst. **a** HRTEM image of Ru_1_Co-SAA at low magnification. Scale bar, 50 nm. **b** HAADF-STEM image of the Ru_1_Co-SAA catalyst. Scale bar, 50 nm. **c** EDS element maps for Co, Ru, Al and O in the dotted-box area. Scale bar, 10 nm. **d** HRTEM image of Ru_1_Co-SAA at high magnification. Scale bar, 5 nm. **e** AC-HAADF-STEM image of Ru_1_Co-SAA at low magnification. Scale bar, 5 nm. **f**, **g** AC-HAADF-STEM image and its color-coded intensity map of Ru_1_Co-SAA at atomic-resolution high magnification. Scale bar, 1 nm. **h** X-Y line profile for Ru and Co atoms, measured from **f**.

The crystallinity of the as-obtained catalysts was studied by powder XRD (Supplementary Fig. 3). The XRD patterns for each sample contained dominant diffraction peaks at around 52° and 61°, corresponding to the (111) and (200) planes of fcc metallic Co. No diffraction peaks due to Ru or RuO_2_ were observed in the XRD patterns of either Ru_1_Co-SAA or Ru_n_Co-NA, indicating that all Ru species were highly dispersed in these materials. Further structural analysis of the catalysts (Supplementary Fig. 4 and Table 2) revealed that Ru_1_Co-SAA, Ru_n_Co-NA and Co-NP catalysts possessed generally similar structures (primarily metallic Co nanoparticles supported on 2D amorphous alumina sheets). High-resolution transmission electron microscopy (HRTEM) images revealed that the Co nanoparticles in Ru_1_Co-SAA possessed an average diameter of 9.8 ± 1.8 nm (Fig. 2a and Supplementary Fig. 5). High-angle annular dark-field scanning transmission electron microscopy (HAADF-STEM) images and energy-dispersive spectroscopy (EDS) element maps (Fig. 2b, c) further confirmed the highly uniform dispersion of Co nanoparticles on amorphous alumina sheets with no Ru nanoparticles present. The lattice fringe spacing on the metal nanoparticles in Ru_1_Co-SAA was 0.20 nm (Fig. 2d), in good accord with the lattice spacing of Co (111). Aberration-corrected high-angle annular dark-field scanning transmission electron microscopy (AC-HAADF-STEM) was able to distinguish single Ru atoms from Co atoms due to differences in the Z-contrast of these elements^39–42^. Figure 2e verified the uniform dispersion of isolated bright Ru atoms in the Co nanoparticles. Furthermore, Fig. 2f, g showed that the single Ru atoms took the place of Co atoms in the lattice. The X-Y line profile over a metal nanoparticle in Ru_1_Co-SAA (Fig. 2h) conclusively proved the presence of Ru atoms in the Co metal lattice, with the Ru atoms having stronger intensity (the 4^th^ and 7^th^ atoms in nine consecutive atoms marked from Fig. 2f)^43,44^. The diameters of Co nanoparticles in the control catalysts (Co-NP and Ru_n_Co-NA, Supplementary Figs. 6-7) were similar to those in the Ru_1_Co-SAA catalyst, though the distribution of Ru in Ru_n_Co-NA was quite different to Ru_1_Co-SAA. For Ru_n_Co-NA, the higher Ru content resulted in a higher Ru coverage on the surface of the Co nanoparticles (Supplementary Fig. 7). A line scan across two adjacent nanoparticles showed that the signals for Ru and Co overlapped, evidence for the formation of a binary RuCo nanoalloy.

**Fig. 3:**
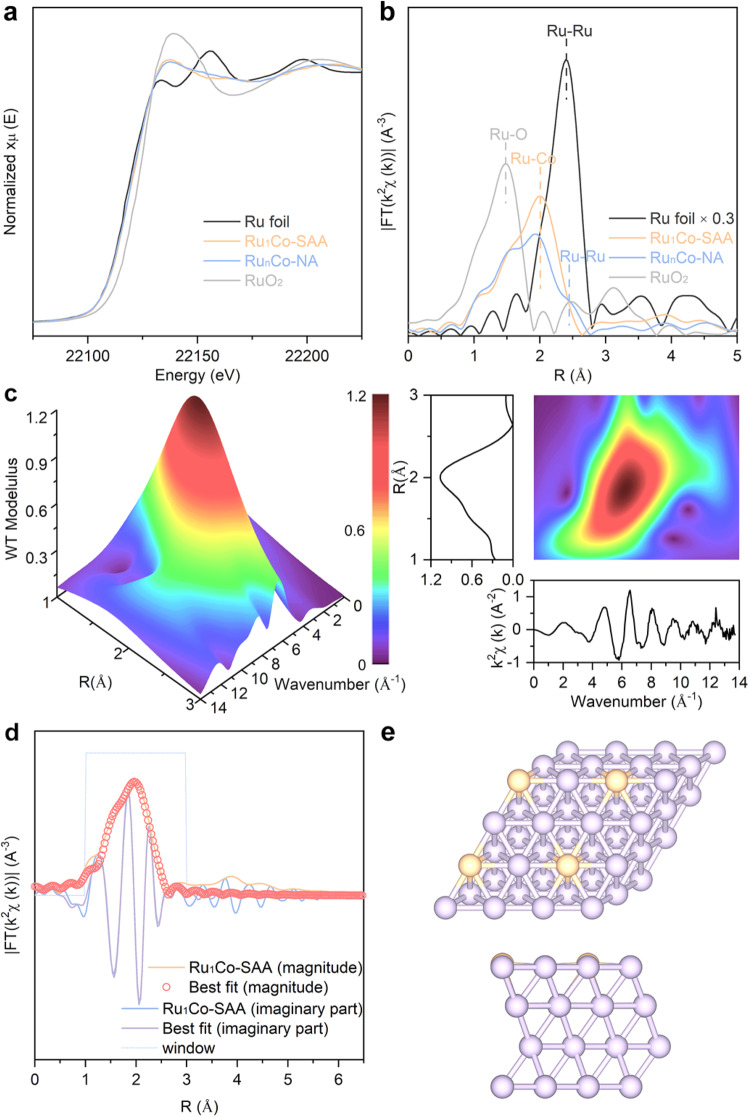
Structural characterization of the Ru_1_Co-SAA catalyst. **a** Ru K-edge XANES for Ru_1_Co-SAA and Ru_n_Co-NA. **b** EXAFS spectra in *R*-space for Ru_1_Co-SAA and Ru_n_Co-NA. **c** WT analysis of Ru_1_Co-SAA. **d** EXAFS fitting curves in *R*-space for Ru_1_Co-SAA. **e** The optimized structure of Ru_1_Co-SAA. Color code: Ru (orange), Co (violet).

Ru K-edge and Co K-edge X-ray absorption spectroscopy (XAS) was next performed to further probe the local structure of the metal nanoparticles in Ru_1_Co-SAA and Ru_n_Co-NA. As shown in Fig. 3a, the Ru K-edge near-edge features of Ru_1_Co-SAA and Ru_n_Co-NA were similar to those of the Ru reference foil, confirming the presence of metallic Ru in the Co nanoparticles. Further analysis in Supplementary Fig. 8 indicated the average oxidation state of Ru in Ru_1_Co-SAA and Ru_n_Co-NA were +0.6 and +1.3, respectively, indicating intra-atomic charge re-distribution in the alloy catalysts. Fourier-transformed *k*^2^-weighted extended X-ray absorption fine structure (EXAFS) in *R*-space allowed elucidation of the coordination environments of Ru atoms in the Co nanoparticles (Fig. 3b). For Ru_1_Co-SAA, a peak at 2.0 Å was observed and assigned to a Ru-Co scattering path (first coordination shell). This feature was conspicuously shorter than the first Ru-Ru coordination shell feature in Ru foil (2.4 Å)^39,45^. No peaks corresponding to the Ru-Ru scattering were observed for Ru_1_Co-SAA, confirming the dominant Ru-Co coordination (i.e. isolated Ru atoms) in the catalyst. In contrast, a distinct shoulder peak at 2.4 Å due to the Ru-Ru scattering was observed for Ru_n_Co-NA. Together with the a weaker Ru-Co coordination than Ru_1_Co-SAA, this indicated the presence of Ru clusters in the RuCo alloy nanoparticles of Ru_n_Co-NA. Wavelet transformed EXAFS (WT-EXAFS) Ru K-edge spectra in *k*-space and *R*-space further revealed the distinct differences between Ru_1_Co-SAA and Ru_n_Co-NA. Ru_1_Co-SAA showed one intensity maximum at the wavenumber 6.5 Å^−1^ in the contour plot (Fig. 3c). In contrast, Ru_n_Co-NA showed another intensity maximum at 9.4 Å^−1^, corresponding to the Ru-Ru region (Supplementary Fig. 9). The Co K-edge XAS data (Supplementary Fig. 10) for the Ru_1_Co-SAA, Ru_n_Co-NA and Co-NP catalysts were similar, revealing a dominant Co-Co scattering path similar to the metallic Co reference foil (evidence for the presence of Co0 nanoparticles rather than oxide phases).

Quantitative chemical configuration analysis of Ru_1_Co-SAA and Ru_n_Co-NA were carried out through the least-squared EXAFS fitting. The *R*-space fitting was performed to estimate the coordination environment of Ru atoms in the first shell (Fig. 3d, Supplementary Fig. 11, and Supplementary Table 3). For Ru_1_Co-SAA, the Ru atoms had an average coordination number of 8.9 and a mean bond length of 2.53 Å between the center Ru atom and surrounding Co atoms. For Ru_n_Co-NA, the Ru-Co coordination number was lower (5.4), whilst the Ru-Ru coordination number was 2.6, demonstrating the existence of metallic Ru-Ru bond (likely as Ru clusters) in Ru_n_Co-NA. The curve-fits in *k* and *q* space revealed a high accuracy of fitting (Supplementary Fig. 12). In the optimized DFT structure for Ru_1_Co-SAA (Fig. 3e), the Ru-Co bond length (2.53 Å) was the same as that determined from the EXAFS fitting, similar to that of Co-Co bond length due to the confinement effect of the fcc Co lattice^31,32^. Furthermore, the results of EXAFS analysis and atomic-resolution AC-HAADF-STEM images for the Ru_1_Co-SAA catalyst were highly consistent, confirming the uniform incorporation of single Ru atoms in the alumina-supported Co nanoparticles.

### CO photo-hydrogenation performance under ambient pressures

**Fig. 4:**
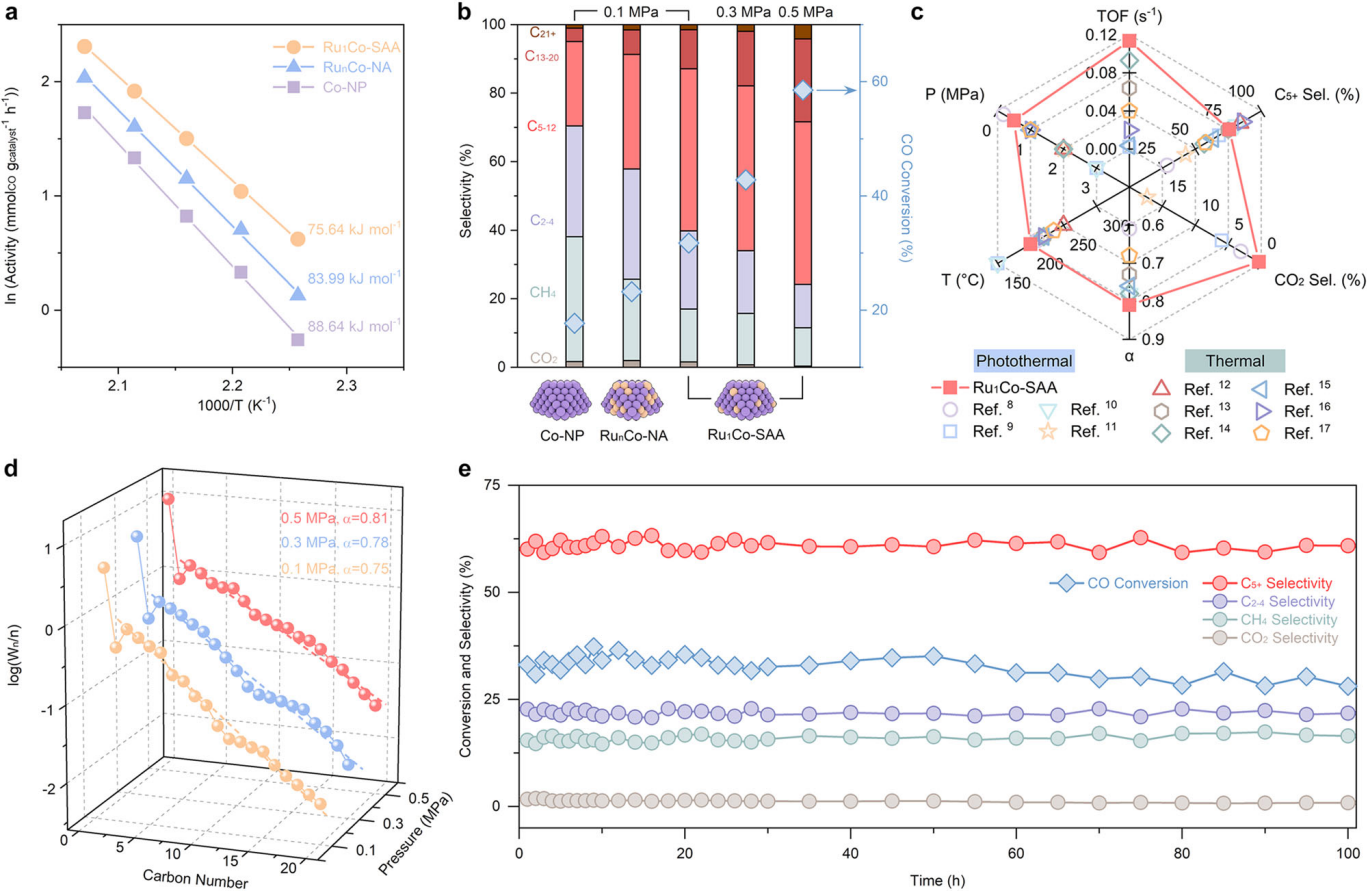
CO photo-hydrogenation performance with different catalysts and reaction conditions. **a** Arrhenius plot for CO conversion activity over Co-NP, Ru_1_Co-SAA and Ru_n_Co-NA. **b** CO photo-hydrogenation performance of Co-NP, Ru_1_Co-SAA and Ru_n_Co-NA (reaction conditions: 50 mg catalyst, 1.80 W cm^−2^ UV-Vis irradiation for 10 h, T = 200 °C, CO/H_2_/N_2_ (20/40/40) as feed gas, GHSV = 2400 mL g^−1^ h^−1^). **c** Comparison of FTS performance of Ru_1_Co-SAA with other state-of-the-art Co-based, Ru-based and RuCo-alloyed catalysts. P pressure, T temperature, α ASF chain growth probability, Sel selectivity, TOF turnover frequency. **d** The hydrocarbon product distribution obtained over Ru_1_Co-SAA under UV-Vis irradiation. **e** Durability test on Ru_1_Co-SAA under 1.80 W cm^−2^ UV-Vis irradiation.

CO photo-hydrogenation tests were carried out in a flow-type system (see Methods for experimental details) to investigate the impact of the RuCo atomic structure on the catalytic performance. The Co-NP, Ru_1_Co-SAA and Ru_n_Co-NA catalysts were all black colored and exhibited strong light absorption across the UV-Vis region (Supplementary Fig. 13). This enabled each catalyst to efficiently convert the photon energy into local heating for driving CO hydrogenation reactions without requiring external heating sources. Under UV-Vis irradiation at a light intensity of 1.80 W cm^−2^, the temperature of Ru_1_Co-SAA catalyst surface rapidly increased to reach 200 °C within 15 min (Supplementary Fig. 14). In order to investigate the photo-hydrogenation activity of catalysts in the flow-type reaction chamber (CO/H_2_/N_2_ = 20/40/40, the gas hourly space velocity, GHSV = 2400 mL g^−1^ h^−1^, 0.1 MPa), the CO conversion was measured at temperature ranging from 170-220 °C (Supplementary Fig. 15). In the measured temperature range, Ru_1_Co-SAA maintained the highest CO conversion amongst the catalysts tested (8.7% at 170 °C to 57.7% at 220 °C). Figure 4a shows apparent activation energies for CO conversion over Co-NP, Ru_1_Co-SAA and Ru_n_Co-NA were 88.64, 75.64, and 83.99 kJ mol^−1^, respectively. Results demonstrate that Ru_1_Co-SAA catalyst had the best activity to drive CO photo-hydrogenation. The C-C coupling of intermediates is an essential process to obtain long-chain liquid fuels in CO hydrogenation. Of particular interest in this context is the C_5+_ selectivity. For the Co-NP catalyst, the CO conversion at 200 °C with atmospheric-pressure syngas was only 17.7%. The main products were C_1-4_ hydrocarbons (68.7% selectivity) and some C_5+_ hydrocarbons (29.5% selectivity), as shown in Fig. 4b and Supplementary Table 4. By introducing the Ru single atom into Co nanoparticles, the C_5+_ selectivity was greatly improved. For Ru_1_Co-SAA, the CO conversion reached 31.8% with a C_5+_ selectivity as high as 60.2%, representing truly outstanding FTS performance under ambient pressure (0.1 MPa). Although Ru_n_Co-NA showed enhanced CO photo-hydrogenation performance compared to the Co-NP catalyst, the promoting effect of Ru was far less pronounced than the Ru_1_Co-SAA catalyst. Results suggest that the Ru-Co coordination in Ru_1_Co-SAA was critical for achieving a high C_5+_ selectivity at low pressures. In addition, the temperature-selectivity relationship for Ru_1_Co-SAA was explored (Supplementary Fig. 16), with a temperature of 200 °C offering the best overall performance in terms of CO conversion with good C_5+_ selectivity. Next, CO photo-hydrogenation experiments at 0.3 and 0.5 MPa were carried out. With the elevation of syngas pressure, the CO conversion over Ru_1_Co-SAA increased to 58.6%, with a TOF of 0.114 s^−1^ and a C_5+_ selectivity of 75.8%. It should be noted that the CO photo-hydrogenation performance of Ru_1_Co-SAA at 0.5 MPa was comparable to best Co-based, Ru-based and RuCo-alloy catalysts operated at much higher pressures (normally above 2 MPa). As illustrated in Fig. 4c and Supplementary Table 5, the performance of Ru_1_Co-SAA ranks superior to all FTS catalysts reported to date when considering six crucial FTS reaction parameters (temperature, pressure, TOF, C_5+_ selectivity, CO_2_ selectivity and chain growth probability α). In particular, Ru_1_Co-SAA exhibited the special advantages of a high TOF, high C_5+_ selectivity and low operating pressure^8–17^. Gas chromatograph (GC) profiles of the gaseous and liquid products formed during CO photo-hydrogenation over Ru_1_Co-SAA are shown in Supplementary Figs. 17, 18. The specific product distributions at various pressures conformed to Anderson-Schulz-Flory (ASF) distributions (Fig. 4d). The α value at reaction pressure of 0.1, 0.3 and 0.5 MPa were determined to be 0.75, 0.78 and 0.81, respectively, indicating that the C-C coupling ability of the Ru_1_Co-SAA catalyst improved as the syngas pressure increased. The comparative thermo-catalytic experiment using only electric heating was performed over Ru_1_Co-SAA, illustrating that the CO photo-hydrogenation followed a photothermal reaction mechanism (Supplementary Fig. 19). The stability of the Ru_1_Co-SAA catalyst was next investigated, with steady CO photo-hydrogenation tests showing no obvious performance losses over 100 h of continuous operation (Fig. 4e). XRD and HADDF-STEM results showed that the structure of the Ru_1_Co-SAA catalyst was unchanged following the stability test (Supplementary Figs. 20, 21).

In order to better understand the catalytic properties of the Co-NP, Ru_1_Co-SAA and Ru_n_Co-NA catalysts, temperature programmed desorption (TPD) measurements were performed. CO-TPD profiles (Supplementary Fig. 22) were collected at a heating rate of 10 °C min^−1^ following CO pre-adsorption at room temperature. The total amounts of adsorbed CO on the Co-NP, Ru_1_Co-SAA and Ru_n_Co-NA catalysts were 2.47, 5.71 and 4.68 mmol g^−1^ respectively, indicating that the addition of Ru significantly enhanced the CO adsorption properties of the Co nanoparticles. The CO desorption peaks at high temperatures originate from the recombinative desorption of surface C* and O* species adsorbed on the metal sites, signifying the strong chemisorption of CO on Ru_1_Co-SAA^13^. The H_2_-TPD profiles (Supplementary Fig. 23) showed that H_2_ adsorption on Ru_1_Co-SAA and Co-NP were very similar, indicating that the single atom Ru sites did not alter the H_2_ adsorption feature of the Co nanoparticles. In contrast, both physical and chemical adsorption of H_2_ were promoted by RuCo alloy nanoparticles in Ru_n_Co-NA. Results demonstrate the addition of atomically dispersed Ru enhanced the chemisorption of CO whilst having negligible impact on H_2_ adsorption.

**Fig. 5:**
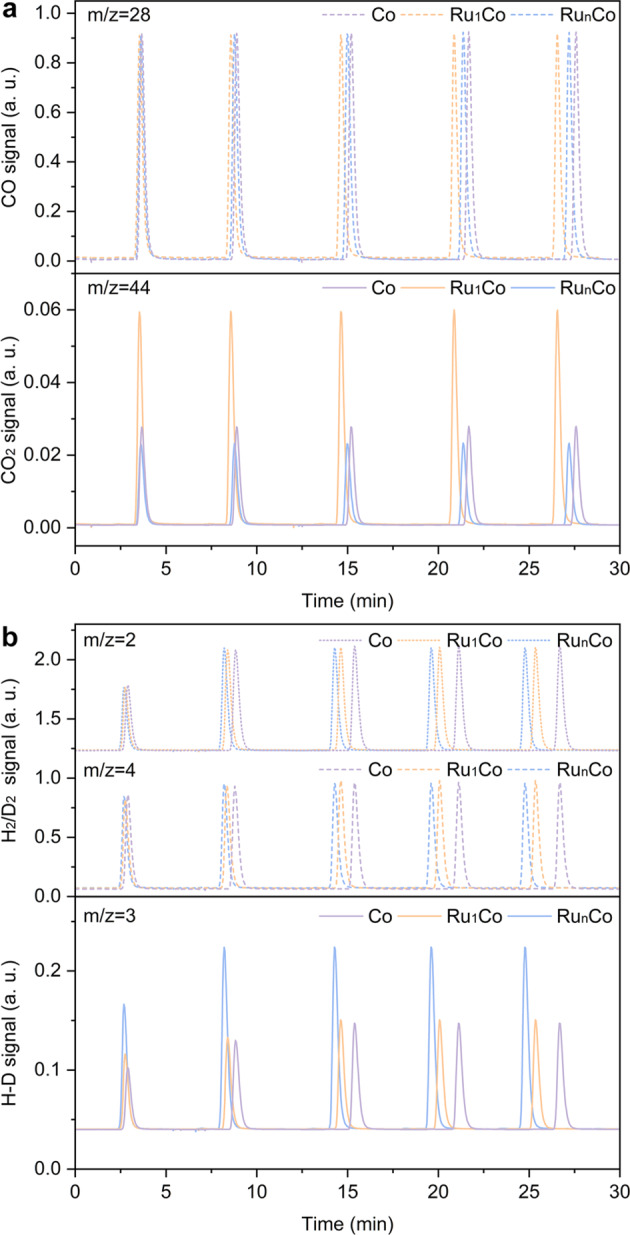
Pulse chemisorption experiments using different catalysts. **a** CO pulse profile with CO (m/z = 28) and CO_2_ (m/z = 44) signals. **b** H_2_ isotopic pulse profile with H_2_ (m/z = 2), D_2_ (m/z = 4) and HD (m/z = 3) signals.

In addition to the surface adsorption of reactants (CO and H_2_), their dissociation on active sites is also an important consideration for efficient CO photo-hydrogenation. To explore this aspect, we conducted pulse chemisorption experiments using both CO pulses and H_2_/D_2_ pulses to evaluate the dissociation ability of the catalyst for CO and H_2_, respectively (with the schematic mechanism shown in Supplementary Fig. 24). All experiments were conducted at 200 °C and ambient pressure (0.1 MPa). Results of the CO pulse experiments are shown in Fig. 5a, which evaluated the ability of the catalysts to activate CO and form C* and O* species on the catalyst surface. The Ru_1_Co-SAA catalyst performed the strongest CO_2_ signal (m/z = 44), indicating that C* and O* species were formed in high coverage from the dissociation of CO^46^. Accordingly, amongst the catalyst studied, Ru_1_Co-SAA offered the best ability for CO conversion. Combining the results of CO-TPD and CO pulse experiments, it can be concluded that the strong interaction between CO and active Ru_1_Co-SAA metal sites (resulting in a long residence time of C* species on the catalyst surface) would promote C-C coupling of CH_x_* intermediates^13,47^. The HD signals (m/z = 3) arising from the H_2_/D_2_ pulse provided valuable information about the hydrogen activation ability of the different catalysts. An excessively high hydrogen activation ability is considered detrimental to the carbon chain growth into C_5+_ liquid fuels in FTS. As shown in Fig. 5b, Ru_1_Co-SAA exhibited a similar HD signal (m/z = 3, representing the hydrogen dissociation ability) to Co-NP, whereas Ru_n_Co-NA showed the strongest HD signal compared to the other catalysts. The strong hydrogen dissociation ability of Ru_n_Co-NA would lead to the over-hydrogenation of CH_x_* intermediates and limit long-chain hydrocarbon growth through C-C coupling reactions^22^. The chemisorption studies demonstrate that atomically dispersed Ru sites in Co nanoparticles enhance the formation of surface C* species without affecting the activation of H_2_, this benefitting C-C coupling reactions and preventing the over-hydrogenation of CH_x_* intermediates.

### DFT studies of CO dissociation, hydrogenation and C-C coupling of CH_x_* intermediates

**Fig. 6:**
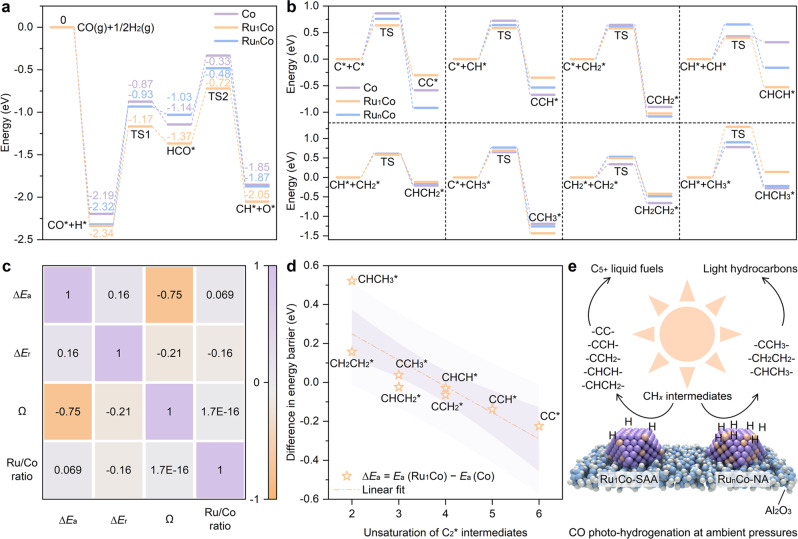
Reaction paths and energy barriers based on DFT calculations. **a** Potential energy profile of CO* dissociation. **b** Potential energy profile of eight C-C coupling paths for C_2_* intermediates. **c** Correlation analysis between the unsaturation of C_2_* intermediates relative to ethane (Ω) and the energy barrier reduction (∆*E*_a_) of Ru_1_Co-SAA minus Co-NP. **d** Linear correlation between unsaturation of C_2_* intermediates relative to ethane (Ω) and energy barrier reduction (∆*E*_a_) of Ru_1_Co-SAA minus Co-NP. **e** Proposed products from intermediates with different unsaturation over Ru_1_Co-SAA and Ru_n_Co-NA through CO photo-hydrogenation at ambient pressures.

To better understand the mechanism by which Ru single atoms promoted CO activation and enhanced long-chain hydrocarbon production, DFT calculations were carried out. The calculation details are provided in the Methods section, with the simulation models shown in Supplementary Fig. 25. According to previous studies, the CO activation via H-assistant path (CO* + H* → HCO* → CH* + O*) is more favorable than direct CO dissociation and other H-assistant pathways^5,48^. Figure 6a shows that the CO adsorption strengths were −2.19 eV on Co (111), −2.34 eV on Ru_1_Co (111), and −2.32 eV on Ru_n_Co (111). The rate determining step in CO activation is the hydrogenation of CO* to form HCO* (CO* + H* → HCO*). The energy barriers (*E*_a_) for this step were similar on the Co (111) and Ru_n_Co (111) surfaces were 1.32 eV and 1.39 eV, respectively, whilst a much lower energy barrier (1.17 eV) existed on the Ru_1_Co (111) surface. From a thermodynamics viewpoint, the H-assistant dissociation of CO* on the Co (111), Ru_1_Co (111) and Ru_n_Co (111) surfaces were exothermic processes, releasing reaction energies (*E*_r_) of −1.85 eV, −2.05 eV, and −1.87 eV, respectively. Therefore, in terms of both kinetics and thermodynamics, CO* dissociation was more favorable on the Ru_1_Co (111) surface compared to the other surfaces. This is consistent with the higher CO conversion over the Ru_1_Co-SAA catalyst by experiment. For the further hydrogenation of CH_x_* intermediates, the Ru_1_Co (111) surface showed similar properties to the Co (111) surface comprehensively considering thermodynamics and kinetics for the steps from CH* to CH_4_ (CH* + 3H* → CH_2_* + 2H* → CH_3_* + H* → CH_4_, see Supplementary Fig. 26 and Supplementary Tables 6-8 for details). In contrast, the Ru_n_Co (111) surface showed lower *E*_a_ and *E*_r_ values compared with the other two catalyst models in almost every step from CH* to CH_4_, demonstrating that Ru-Ru coordination environments in Ru_n_Co-NA caused over-hydrogenation of CH_x_* intermediates.

For C-C coupling reactions on the three surface models, eight possible paths for producing C_2_* intermediates were considered (Fig. 6b and Supplementary Fig. 27). The *E*_a_ and *E*_r_ values for these different paths were summarized in Supplementary Tables 9–11. For five paths (in which C_2_* species of CC*, CCH*, CCH_2_*, CHCH* and CHCH_2_* were generated), the Ru_1_Co (111) surface offered the lowest C-C coupling *E*_a_ (Supplementary Fig. 28). For two paths (those leading to CHCH* and CCH_3_*), the Ru_1_Co (111) surface offered the lowest C-C coupling *E*_r_. Considering that the CO photo-hydrogenation tests in the current work were performed at ambient and near ambient pressures, *E*_a_ is the main metric of interest since there was no requirement to apply high pressures to overcome the C-C coupling energy barriers (as is often the case in thermal FTS reactions to higher liquid hydrocarbons). In comparison, the Ru_n_Co (111) surface offered the second lowest *E*_a_ in five C-C coupling paths (leading to CC*, CCH*, CCH_2_*, CHCH_2_*, and CHCH_3_*), whilst the Co (111) surface had the highest *E*_a_ in four C-C coupling paths (leading to CC*, CCH*, CCH_2_*, and CHCH_2_*). The low *E*_a_ values for C-C coupling reactions on the model Ru_1_Co (111) surface suggested that Ru-Co site coordination possessed higher intrinsic C-C coupling ability compared to Co-Co and Ru-Ru coordination environments.

## Discussion

For deeper statistical analysis of C-C coupling processes on the various catalytic surfaces, four factors were considered to obtain the Pearson correlation coefficients: the difference in energy barriers relative to Co-NP (∆*E*_a_), the difference in reaction energy relative to Co-NP (∆*E*_r_), the unsaturation of C_2_* intermediates relative to ethane (Ω, see the detailed calculation in Methods) and Ru/Co ratio of the model surfaces. The results of correlation analysis are shown in Fig. 6c and Supplementary Table 12. The correlation coefficient between ∆*E*_a_ and Ω (−0.75, approaching −1) indicated a strong and negative correlation. The p-value (0.0008) between ∆*E*_a_ and Ω was much lower than 0.05, indicating the significance of their correlation coefficient. In contrast, the absolute value of the other correlation coefficients was no higher than 0.21, demonstrating that the influence of Ω and Ru/Co ratios on ∆*E*_r_ were not significant. Moreover, the C_5+_ selectivity in CO photo-hydrogenation experiments correlated with the kinetics of C_2_* intermediate formation. This suggests that *E*_a_ represents an important index when evaluating the intrinsic C-C coupling ability of FTS catalytic surfaces. As shown in Fig. 6d, ∆*E*_a_ decreased linearly with an increase in unsaturation, Ω. The negative ∆*E*_a_ values reflect the lower *E*_a_ of C-C coupling paths for C_2_* intermediates with high Ω (CC*, CCH*, CCH_2_*, CHCH* and CHCH_2_*) on Ru_1_Co (111) relative to Co (111). In contrast, the formation for C_2_* species with low Ω (CHCH_3_*, CH_2_CH_2_*, and CCH_3_*) are inhibited, as indicated by the more positive ∆*E*_a_ relative to Co (111). Considering that the hydrogenation and C-C coupling of unsaturated intermediates can be regarded as competitive reactions, C_2_* species with low Ω tend to be over-hydrogenated and desorb as light hydrocarbons, whereas C_2_* intermediates with high Ω are more likely to undergo carbon chain growth to produce long-chain products. Combining the hydrogenation and coupling processes of CH_x_* species, highly unsaturated C_2_* intermediates were generated over Ru_1_Co-SAA for further C-C coupling to C_5+_ liquid fuels (Fig. 6e). By comparison, relatively saturated intermediates over Ru_n_Co-NA limited the C-C coupling and were easily over-hydrogenated to light hydrocarbons. Hence, Ru_1_Co-SAA exhibited an unusually high selectivity for long-chain hydrocarbons at atmospheric pressure, whereas Co-NP and Ru_n_Co-NA tended to produce light hydrocarbons under the same testing conditions.

In summary, an atomically dispersed Ru_1_Co-SAA catalyst was obtained from an LDH precursor which demonstrated outstanding activity for CO photo-hydrogenation to C_5+_ liquid fuels at ambient pressures. The presence of atomically dispersed Ru atoms on the Co nanoparticles in Ru_1_Co-SAA catalyst enhanced CO activation and lowered the energy barrier for C-C coupling reactions without adversely promoting H_2_ activation and the further CH_x_* over-hydrogenation. Owing to these attributes, Ru_1_Co-SAA exhibited an outstanding CO conversion (58.6%), TOF (0.114 s^−1^), C_5+_ selectivity (75.8%) and remarkable stability (100 h with no deterioration) in flow-type CO photo-hydrogenation reaction at ambient pressures. CO and H_2_ chemisorption experiments and density functional theory calculations further confirmed the key role of Ru single atoms (Ru-Co coordination) in promoting CO activation and C-C coupling reactions, whilst suppressing CH_x_* hydrogenation processes that yield light hydrocarbons. This work illustrates the untapped potential of single atom alloy catalysts in photothermal FTS, whilst providing a framework for the rational future design of FTS catalysts that yield valuable liquid fuels at extremely mild pressures (including atmospheric pressure).

## Methods

### Materials

Co(NO_3_)_2_·6H_2_O, Al(NO_3_)_3_·9H_2_O and hexamethylenetetramine (HMT) were obtained from Beijing Chemical Works (Beijing, China). RuCl_3_·3H_2_O was obtained from Beijing Innochem Science & Technology Co., Ltd. Syngas (mole ratio CO/H_2_/N_2_ = 20/40/40) was purchased from Beijing SIDADE RM Science and Technology Co., Ltd. All materials were used without further purification. Deionized water was used in the synthesis of all catalysts.

### Synthesis of LDH precursors

Ru_1_CoAl-LDH and Ru_n_CoAl-LDH were synthesized via a simple one-pot hydrothermal method. Briefly, Co(NO_3_)_2_·6H_2_O (0.005 mol), Al(NO_3_)_3_·9H_2_O (0.005 mol), HMT (0.013 mol) and RuCl_3_·3H_2_O (amount used depending on the desired Ru/Co molar ratio in the LDH product) were dissolved in deionized water (40 mL). Then the mixed metal-salt solution was heated at 120 °C for 24 h in a Teflon-lined stainless-steel autoclave. After cooling to room temperature naturally, the obtained precipitate was centrifugated and washed 3 times with deionized water. After drying at 60 °C for 12 h, the LDH products were ground to fine powders with a mortar and pestle. CoAl-LDH was prepared by the same method without adding RuCl_3_·3H_2_O.

### Synthesis of Ru_1_Co-SAA, Ru_n_Co-NA and Co-NP catalysts

The LDH powders were reduced in a H_2_/Ar (10/90 v/v) flow at 650 °C for 300 min, using a heating rate of 5 °C min^−1^. The products obtained from Ru_1_CoAl-LDH, Ru_n_CoAl-LDH and CoAl-LDH are denoted herein as Ru_1_Co-SAA, Ru_n_Co-NA and Co-NP, respectively. Following the reduction step, the catalysts were allowed to cool naturally to room temperature in the H_2_/Ar flow, then stored at room temperature under a N_2_ atmosphere.

### Characterization

XRD patterns were collected on a Bruker DAVINCI D8 ADVANCE diffractometer. Sample morphologies (HRTEM, HAADF-STEM and EDS element maps) were examined using a JEOL-2100F. Aberration-corrected HAADF-STEM images were conducted on a Titan Themis G2 instrument. Nitrogen adsorption-desorption isotherms were collected at 77 K on a Quantachrome Quadrasorb SI-MP instrument. Brunauer-Emmett-Teller specific surface areas, total pore volumes and average pore diameters and pore size distributions were calculated from the N_2_ physisorption isotherms. UV-Vis diffuse reflectance spectra were collected on a Cary 7000 instrument equipped with an integrating sphere attachment. Inductively coupled plasma-optical emission spectroscopy (ICP-OES, Varian 710) was used to quantify the amounts of Co and Ru in the samples. X-ray absorption spectroscopy (XAS) measurements were performed at the Beijing Synchrotron Radiation Facility (Beamline 1W1B). H_2_-TPR, CO-TPD and H_2_-TPD data were acquired on a Micromeritics AutoChem II 2920 instrument equipped with a thermal conductivity detector (TCD). Pulse chemisorption experiments were conducted on a Micromeritics AutoChem II 2920 instrument equipped with a TCD and a Hiden QIC-20 mass spectrometer.

### CO photo-hydrogenation tests

CO hydrogenation tests were carried out in a flow-type reaction chamber with a quartz window at the top for catalyst irradiation^5–8,49,50^. Briefly, 50 mg of catalyst was uniformly spread as a thin layer in the reaction chamber, covering the temperature probe. Before UV-Vis irradiation, syngas (2 mL min^−1^, CO/H_2_/N_2_ = 20/40/40) was flowed through the reaction chamber for 30 min to remove any air. Subsequently, the flow-type CO photo-hydrogenation reaction was initiated by light irradiation (300 W Xe lamp, Beijing Perfectlight Technology Co. Ltd, PLS-SXE300D, 200 nm < λ <800 nm). The gas products (C_1_ to C_6_) were detected and quantified every hour by a GC (Shimadzu GC-2014C, Shimadzu Co. Japan) equipped with three columns and three detectors used for product analysis (see our previous work for full details)^5–8,49,50^. The liquid products were detected and quantified by a GC (Shimadzu GC-2014) equipped with a flame ionization detector (FID) and an HP-5 column. The CO conversion (CO Con.) was calculated as follows:

1 $${{{{{\rm{CO}}}}}}\,{{{{{\rm{Con}}}}}}.=\frac{{{{{{{\rm{CO}}}}}}}_{{{{{{\rm{in}}}}}}}-{{{{{{\rm{CO}}}}}}}_{{{{{{\rm{out}}}}}}}}{{{{{{{\rm{CO}}}}}}}_{{{{{{\rm{in}}}}}}}}\times 100 \%=\frac{\frac{{{{{{{\rm{A}}}}}}}_{{{{{{\rm{CO}}}}}},{{{{{\rm{in}}}}}}}}{{{{{{{\rm{A}}}}}}}_{{{{{{\rm{N2}}}}}},{{{{{\rm{in}}}}}}}}-\frac{{{{{{{\rm{A}}}}}}}_{{{{{{\rm{CO}}}}}},{{{{{\rm{out}}}}}}}}{{{{{{{\rm{A}}}}}}}_{{{{{{\rm{N2}}}}}},{{{{{\rm{out}}}}}}}}}{\frac{{{{{{{\rm{A}}}}}}}_{{{{{{\rm{CO}}}}}},{{{{{\rm{in}}}}}}}}{{{{{{{\rm{A}}}}}}}_{{{{{{\rm{N2}}}}}},{{{{{\rm{in}}}}}}}}}\times 100 \% $$

where CO_in_ and CO_out_ are the moles of CO at the reactor inlet and the outlet, respectively; A_CO, in_ and A_N2, in_ are the chromatographic peak areas of CO and N_2_ detected by TCD in the feed gas, and A_CO, out_ and A_N2, out_ are the chromatographic peak areas of CO and N_2_ detected by TCD in the product gas stream.

The CO conversion rate (in units of mmol g_cat_^−1^ h^−1^) was calculated as:

2 $${{{{{\rm{CO}}}}}}\,{{{{{\rm{conversion}}}}}}\,{{{{{\rm{rate}}}}}}=\frac{{{{{{\rm{GHSV}}}}}}\times {{{{{\rm{CO}}}}}}\,{{{{{\rm{Con}}}}}}.\times {{{{{\rm{CO}}}}}}\,{{{{{\rm{concentration}}}}}}}{22.4}$$

where GHSV is the gas hourly space velocity (2400 mL g^−1^ h^−1^) and CO concentration is 20.15% in the feed gas.

The TOF (in units of moles CO per mole of metal at the surface per second, abbreviated to s^−1^) was determined using the following equation^19^:

3 $${{{{{\rm{TOF}}}}}}=\frac{{{{{{{\rm{F}}}}}}}_{{{{{{\rm{CO}}}}}}}\times {{{{{\rm{CO}}}}}}\,{{{{{\rm{Con}}}}}}.}{{{{{{{\rm{n}}}}}}}_{{{{{{\rm{s}}}}}}}}$$

where F_CO_ is the moles of CO in flow gas per second; n_s_ is the number of active sites on the catalyst surface measured using hydrogen chemisorption^19,51^.

The product selectivity to C_n_H_m_ and CO_2_ were calculated as:

4 $${{{{{{\rm{C}}}}}}}_{{{{{{\rm{n}}}}}}}{{{{{{\rm{H}}}}}}}_{{{{{{\rm{m}}}}}}}\,{{{{{\rm{Sel}}}}}}.=\frac{{{{{{{\rm{nC}}}}}}}_{{{{{{\rm{n}}}}}}}{{{{{{\rm{H}}}}}}}_{{{{{{\rm{m}}}}}}}}{{{{{{{\rm{CO}}}}}}}_{2}+{\sum }_{{{{{{\rm{n}}}}}}=1}^{{{\max }}}{{{{{{\rm{nC}}}}}}}_{{{{{{\rm{n}}}}}}}{{{{{{\rm{H}}}}}}}_{{{{{{\rm{m}}}}}}}}\times 100 \% $$

5 $${{{{{{\rm{CO}}}}}}}_{2}\,{{{{{\rm{Sel}}}}}}.=\frac{{{{{{{\rm{CO}}}}}}}_{2}}{{{{{{{\rm{CO}}}}}}}_{2}+{\sum }_{{{{{{\rm{n}}}}}}=1}^{{{\max }}}{{{{{{\rm{nC}}}}}}}_{{{{{{\rm{n}}}}}}}{{{{{{\rm{H}}}}}}}_{{{{{{\rm{m}}}}}}}}\times 100 \% $$

where C_n_H_m_ is the moles of hydrocarbons generated in the 10-hour CO photo-hydrogenation reaction, in which n refers to the carbon number from 1 to 20+ detected by FID; CO_2_ refers to the moles of CO_2_ detected by TCD in the off-gas. The carbon balance calculated for all catalysts under different reaction conditions was greater than 90% in this study. Specifically, the carbon balance was 96.20% over Ru_1_Co-SAA at 0.5 MPa, calculated from the consumption rate of CO (12.54 mmol_C_ g_cat_^−1^ h^−1^) and the measured production rate of carbon in the products (12.07 mmol_C_ g_cat_^−1^ h^−1^ including 4.30 mmol_C_ g_cat_^−1^ h^−1^ of C_1-6_ products and 7.77 mmol_C_ g_cat_^−1^ h^−1^ of C_7-30_ products).

The Anderson-Schulz-Flory (ASF) chain growth probability (α) for the FTS were calculated according to the following equation^13,52^:

6 $${{{{{{\rm{W}}}}}}}_{{{{{{\rm{n}}}}}}}={{{{{\rm{n}}}}}}\times {\alpha }^{{{{{{\rm{n}}}}}}-1}\times {(1-\alpha )}^{2}$$

where n is the carbon number of each product from C_1_ to C_20_; W_n_ is the weight fraction of products containing n carbon atoms; and 1 − α is the probability of chain termination^14^.

### CO pulse and H_2_ isotopic pulse experiments

Typically, the as-obtained catalysts (Ru_1_Co-SAA, Ru_n_Co-NA and Co-NP, 100 mg) were heated to 650 °C at a rate of 10 °C min^−1^ under a H_2_/He (10%/90%) atmosphere for 1 h to pre-reduce the catalysts. Then, the temperature was decreased to 200 °C and any remaining H_2_ purged using a high-purity He gas flow until the baseline was stable. CO pulses using a CO/He (10%/90%) gas mixture were then performed 5 times, with the CO (m/z = 28) and CO_2_ (m/z = 44) signals being monitored using a TPD-MS detector. The CO_2_ signal originated from the recombination of *C and *O species which were generated by the chemisorption of CO on catalysts at 200 °C. For the H_2_/D_2_ pulse experiments, the same pre-reduction process was performed, followed by H_2_/D_2_ pulses using a H_2_/D_2_/He (5%/5%/90%) gas mixture for 5 times. The H_2_ (m/z = 2), HD (m/z = 3) and D_2_ (m/z = 4) signals were monitored using a TPD-MS detector. The HD signal originated from the recombination of *H and *D species arising from the chemisorption of H_2_ and D_2_ on the catalysts at 200 °C.

### Computational models

A four-atom-layer Co (111) with a (4 × 4) supercell was used to represent the Co-NP catalyst model (Supplementary Fig. 25). For the Ru_1_Co-SAA and Ru_n_Co-NA catalyst models, nonadjacent and adjacent surface cobalt atoms were replaced with ruthenium atoms, respectively (Supplementary Fig. 25). The Co (111) model contained 64 Co atoms, the Ru_1_Co (111) model contains 4 Ru atoms and 60 Co atoms, whilst the Ru_n_Co (111) model contained 8 Ru atoms and 56 Co atoms. In geometrical optimization calculations, the bottom two layers were fixed, and the top two layers were allowed to relax. A Gamma-centered (2 × 2 × 1) K mesh was used for all slab models. A vacuum layer of 15 Å was applied to avoid the interactions between slabs in the z direction.

### Computational methods

Spin-polarized calculations were performed using the Vienna Ab Initio Simulation Package (VASP) and employing the frozen-core projector-augmented wave (PAW) method. The generalized gradient approximation in the Perdew-Burke-Ernzerhof (GGA-PBE) function was used for the exchange-correlation energy. A cutoff energy of 400 eV was selected for the plane-wave expansion. The convergence criteria for the force and electronic self-consistent iteration were set to 0.05 eV/Å and 10^−4^ eV, respectively. In all calculations, adsorption energies (*E*_ads_) were calculated based on *E*_ads_ = *E*_x/slab_ *–* *[E*_slab_ + *E*_x_*]*, where *E*_x/slab_ is the total energy of the slab with adsorbents after full relaxation, *E*_slab_ is the total energy of the bare slab, and *E*_x_ is the total energy of the free adsorbents in the gas phase. Therefore, the more negative the *E*_ads_, the stronger the adsorption. Reaction energies (*E*_r_) were defined as *E*_r_ = *E*_final_ − *E*_initial_, where *E*_final_ and *E*_initial_ represents the final state energy and initial state energy, respectively. Therefore, a negative *E*_r_ represents an exothermic process. The energy barrier (*E*_a_) was calculated by *E*_a_ = *E*_trans_ − *E*_initial_, where *E*_trans_ and *E*_initial_ represents the transition state energy and initial state, respectively. All transition states were calculated using the climbing image nudged elastic band method (CI-NEB), with the stretching frequencies analyzed in order to characterize whether a stationary point is a minimum state without an imaginary frequency or a transition state with only one imaginary frequency.

The unsaturation of C_2_* intermediates relative to ethane (Ω) was calculated according to the following equation:

7 $$\Omega=6-{{{{{\rm{y}}}}}}$$

where y is the hydrogen number of C_2_* intermediates. For the saturated C_2_ product (ethane, C_2_H_6_), Ω is 0.

## Supplementary information

Supplementary Information

## Data Availability

The datasets generated and/or analyzed during the current study are available from the corresponding author on reasonable request. Source data are provided with this paper. Received: ((will be filled in by the editorial staff)) Accepted: ((will be filled in by the editorial staff)) Published online: ((will be filled in by the editorial staff))
