# Supplementary material for: Ruthenium-cobalt single atom alloy for CO photo-hydrogenation to liquid fuels at ambient pressures
Source: Nat Commun. 2023 Apr 5;14:1909. doi: 10.1038/s41467-023-37631-5 (PMC10076290; doi:10.1038/s41467-023-37631-5)
Supplement: Supplementary file 1 — Supplementary Information [file 41467_2023_37631_MOESM1_ESM.pdf]

Supplementary Information

Ruthenium-cobalt single atom alloy for CO photo-hydrogenation

to liquid fuels at ambient pressures

Zhao et al.

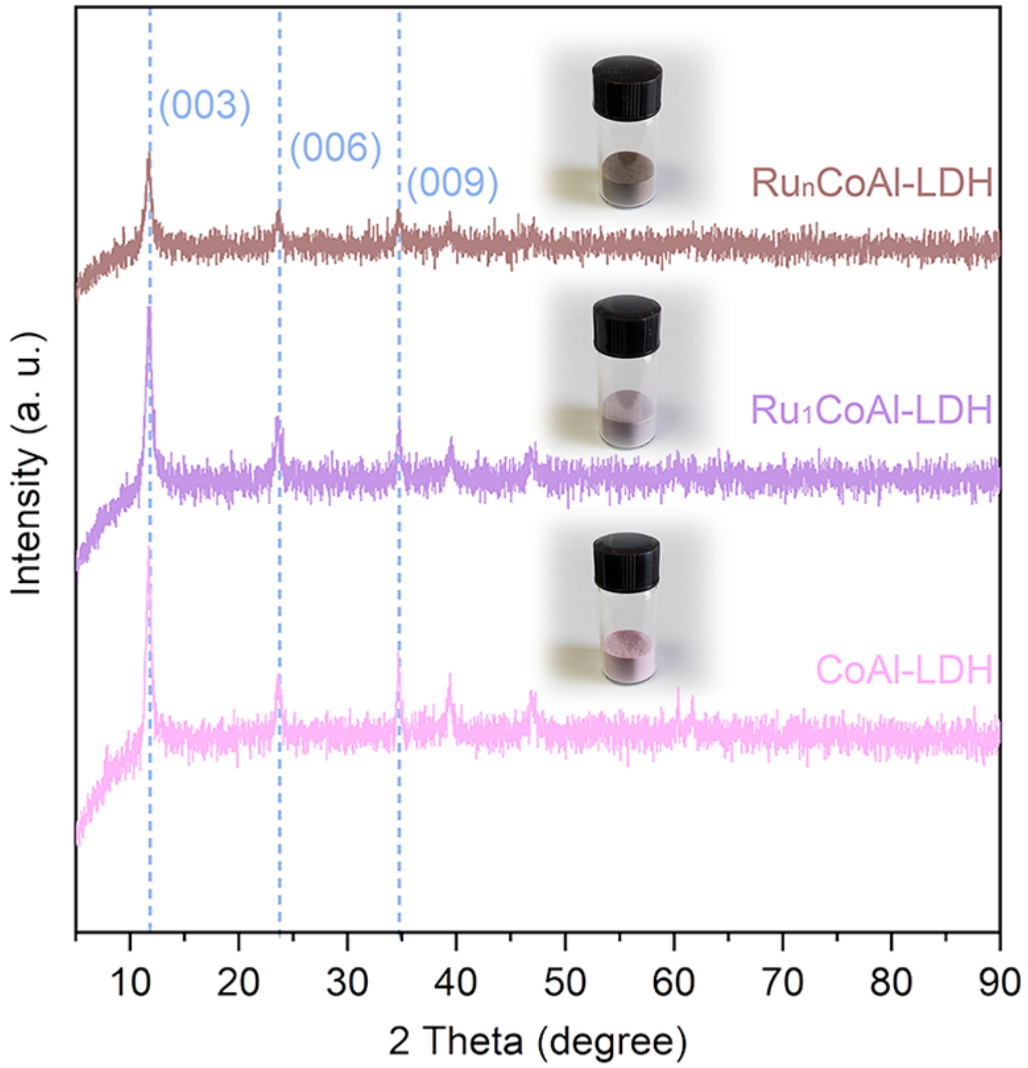

Supplementary Figure 1. XRD patterns and digital photographs for CoAl-LDH, Ru_1CoAl-LDH and Ru_nCoAl-LDH.

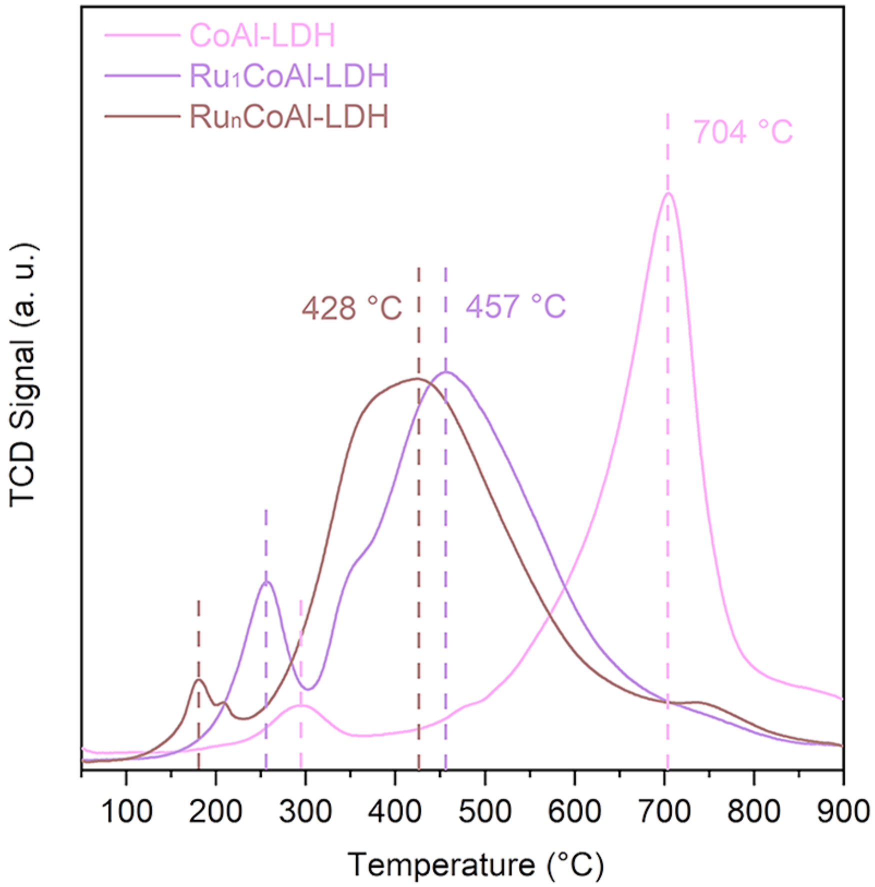

Supplementary Figure 2. H_2-TPR profiles for CoAl-LDH, Ru_1CoAl-LDH and Ru_nCoAl-LDH.

The addition of Ru significantly decreased the reduction temperature for the topological transformation of the LDHs to metal oxides and also metal nanoparticle evolution. The minor peaks below 350 ^{o}C correspond to the dehydroxylation of LDH nanosheets to metal oxides. The main peaks at 350-700 ^{o}C arise from the reduction of cobalt oxides and Ru-containing cobalt oxides to metallic nanoparticles.

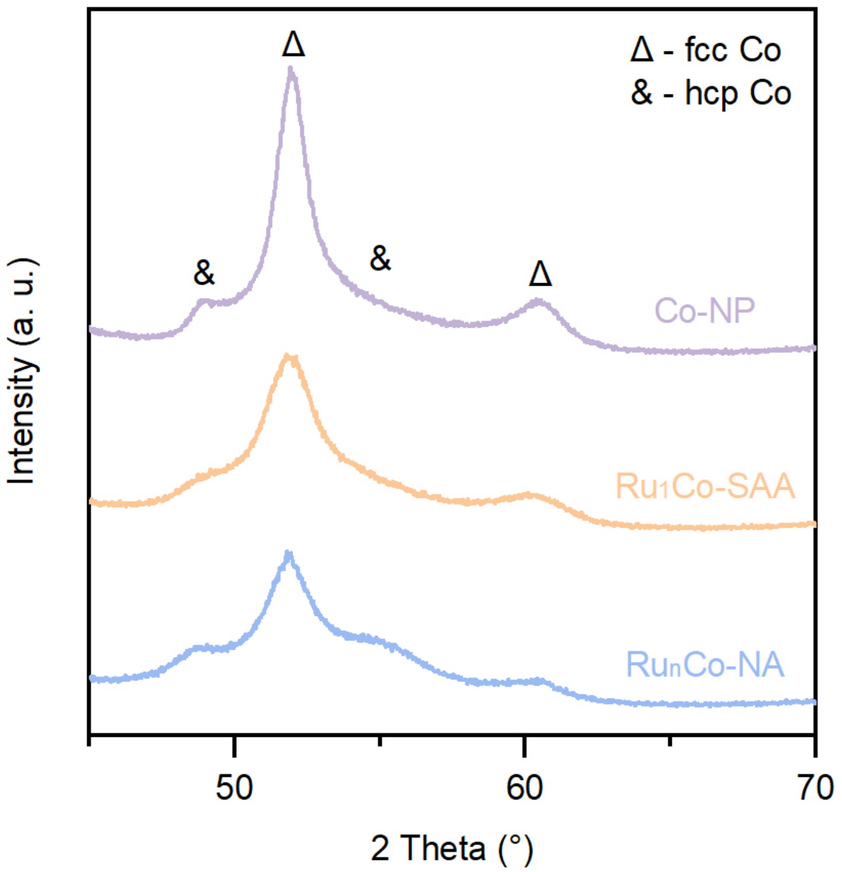

Supplementary Figure 3. XRD patterns for the Co-NP, Ru_1Co-SAA and Ru_nCo-NA catalysts obtained by H_2-reduction of CoAl-LDH, Ru_1CoAl-LDH and Ru_nCoAl-LDH, respectively.

Peaks due to fcc Co were dominant in all the catalysts along with a very small amount of hcp Co, consistent with the HRTEM results.

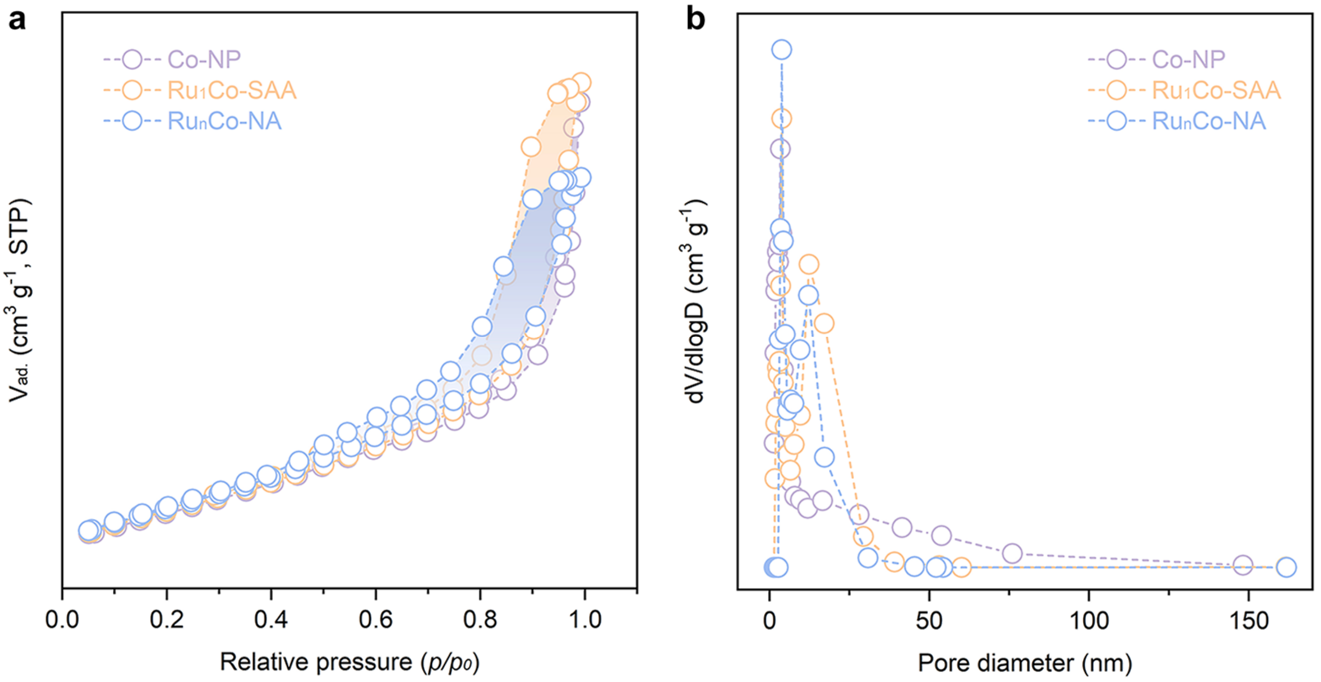

Supplementary Figure 4. (a) N_2 adsorption-desorption isotherms and (b) pore size distributions for the Co-NP, Ru_1Co-SAA and Ru_nCo-NA catalysts.

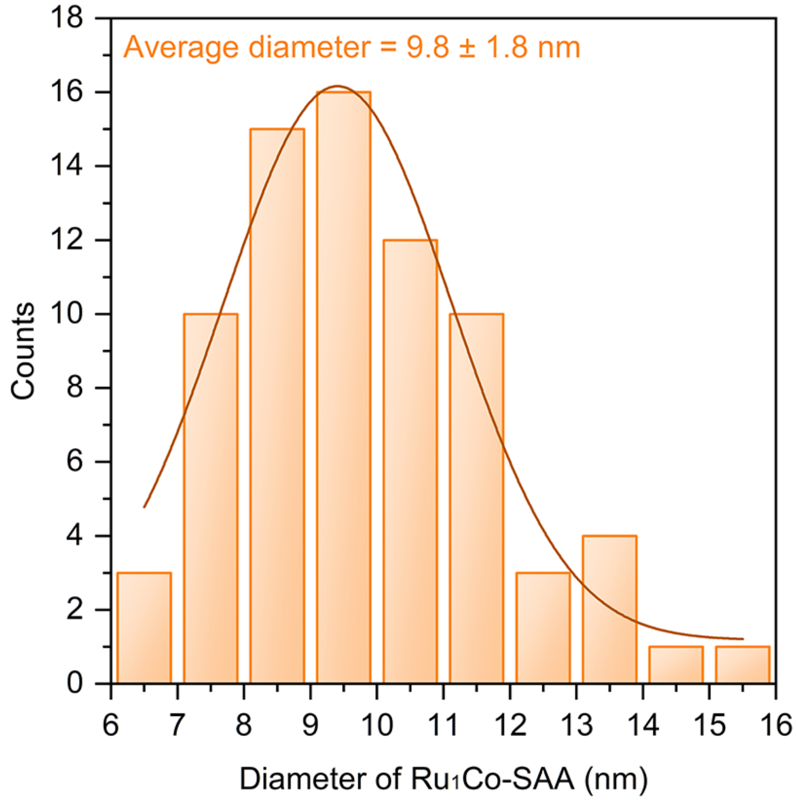

Supplementary Figure 5. Ru_1Co nanoparticle size distribution in the Ru_1Co-SAA catalyst.

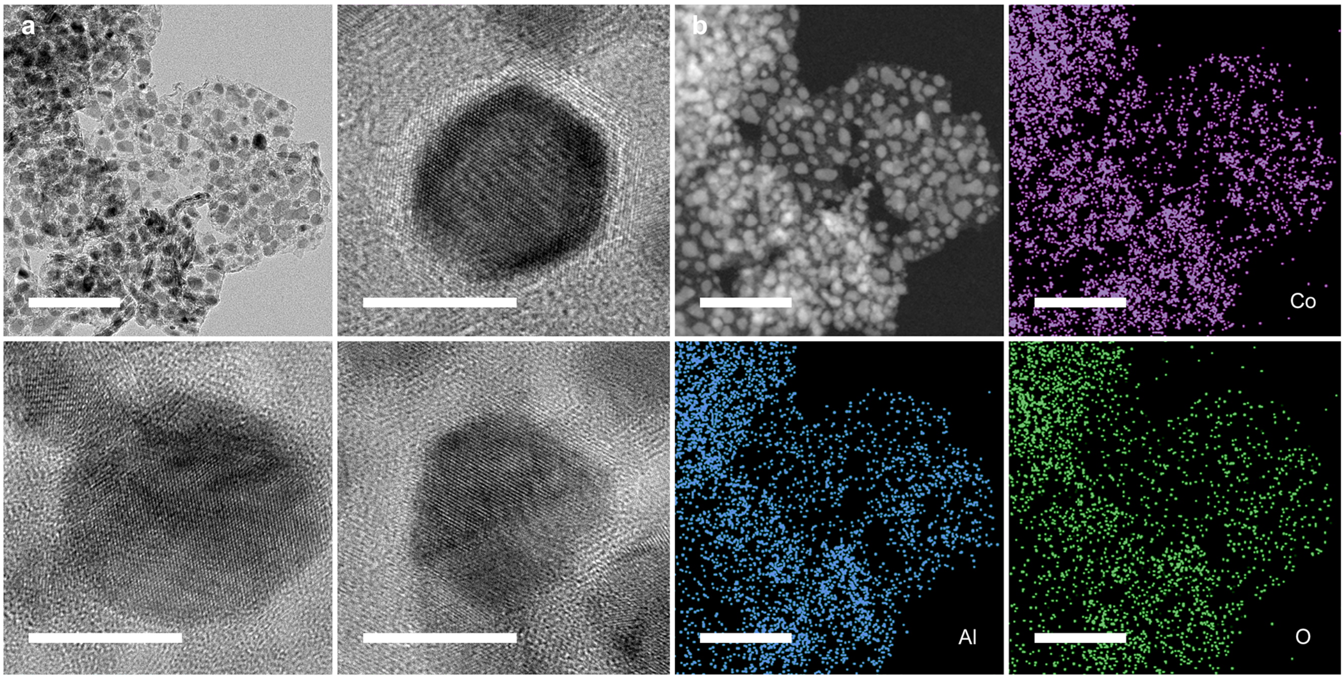

Supplementary Figure 6. (a) HRTEM images of the Co-NP catalyst. Scale bar, 100 nm at low magnification and 10 nm at high magnification. (b) HAADF-STEM image and EDS element maps of Co, Al and O. Scale bar, 100 nm.

The Co-NP catalyst derived from CoAl-LDH possessed a similar morphology to Ru_1Co-SAA, comprising Co nanoparticles of average size ~10 nm supported on 2D amorphous alumina sheets.

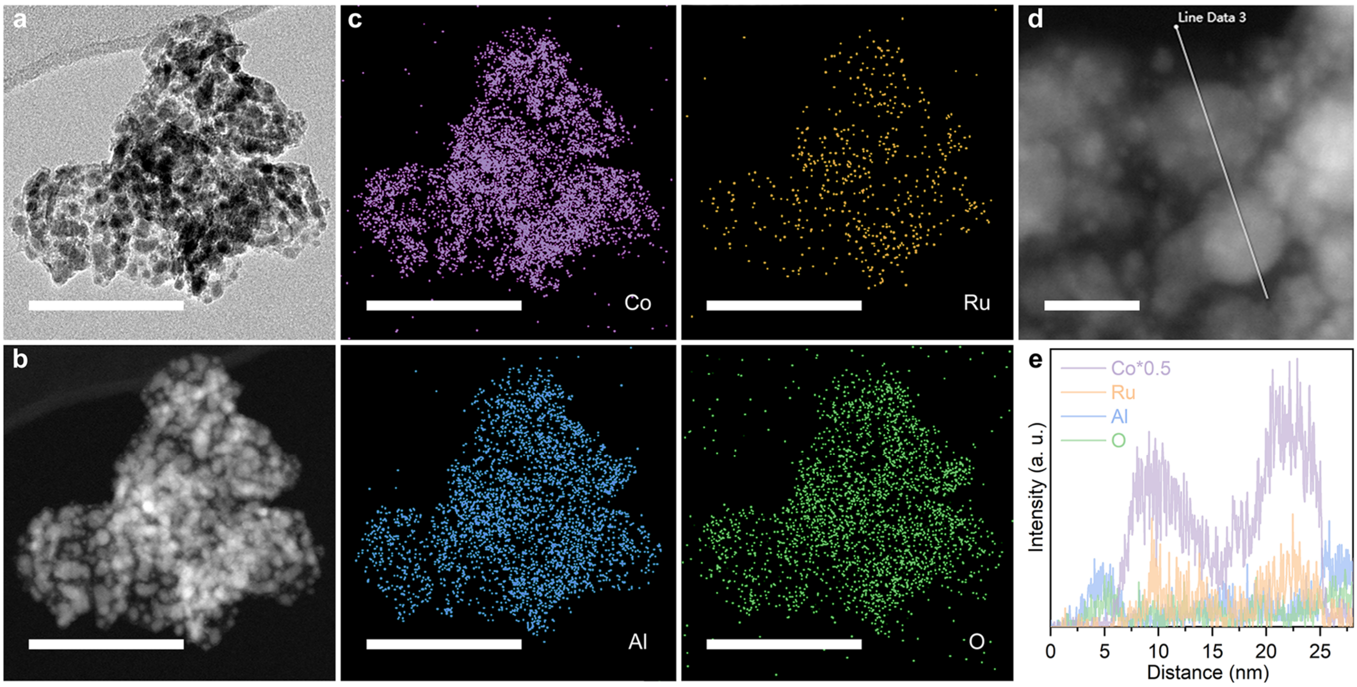

Supplementary Figure 7. (a) HRTEM image, (b) HAADF-STEM image and (c) EDS element maps for the Ru_nCo-NA catalyst. Scale bar, 100 nm. (d) Line-scan image across two neighboring metal nanoparticles. Scale bar, 10 nm. (e) Line-scan EDS profile.

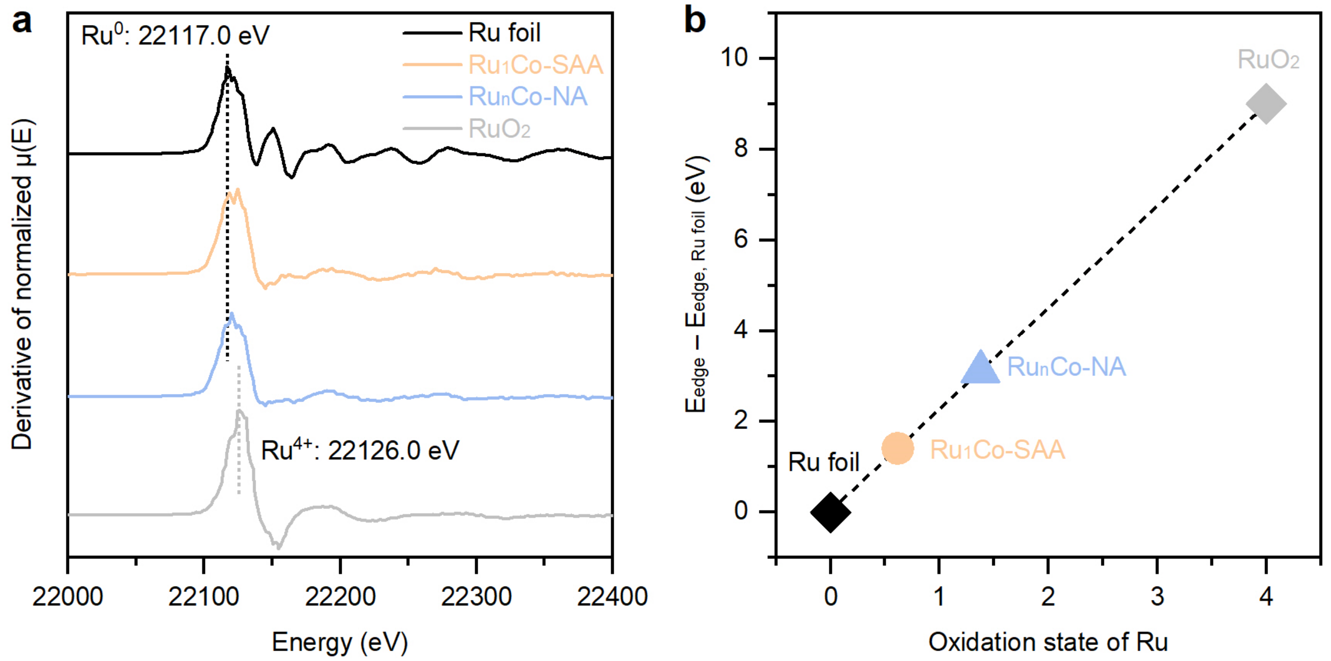

Supplementary Figure 8. (a) Derivative of normalized μ(E) for the Ru_1Co-SAA and Ru_nCo-NA catalysts, and selected reference materials. (b) Oxidation state of Ru calculated form the absorption edge energy in (a).

The oxidation state of Ru in Ru_1Co-SAA and Ru_nCo-NA were between Ru^0 and Ru^{4+}, but much closer to Ru^0.

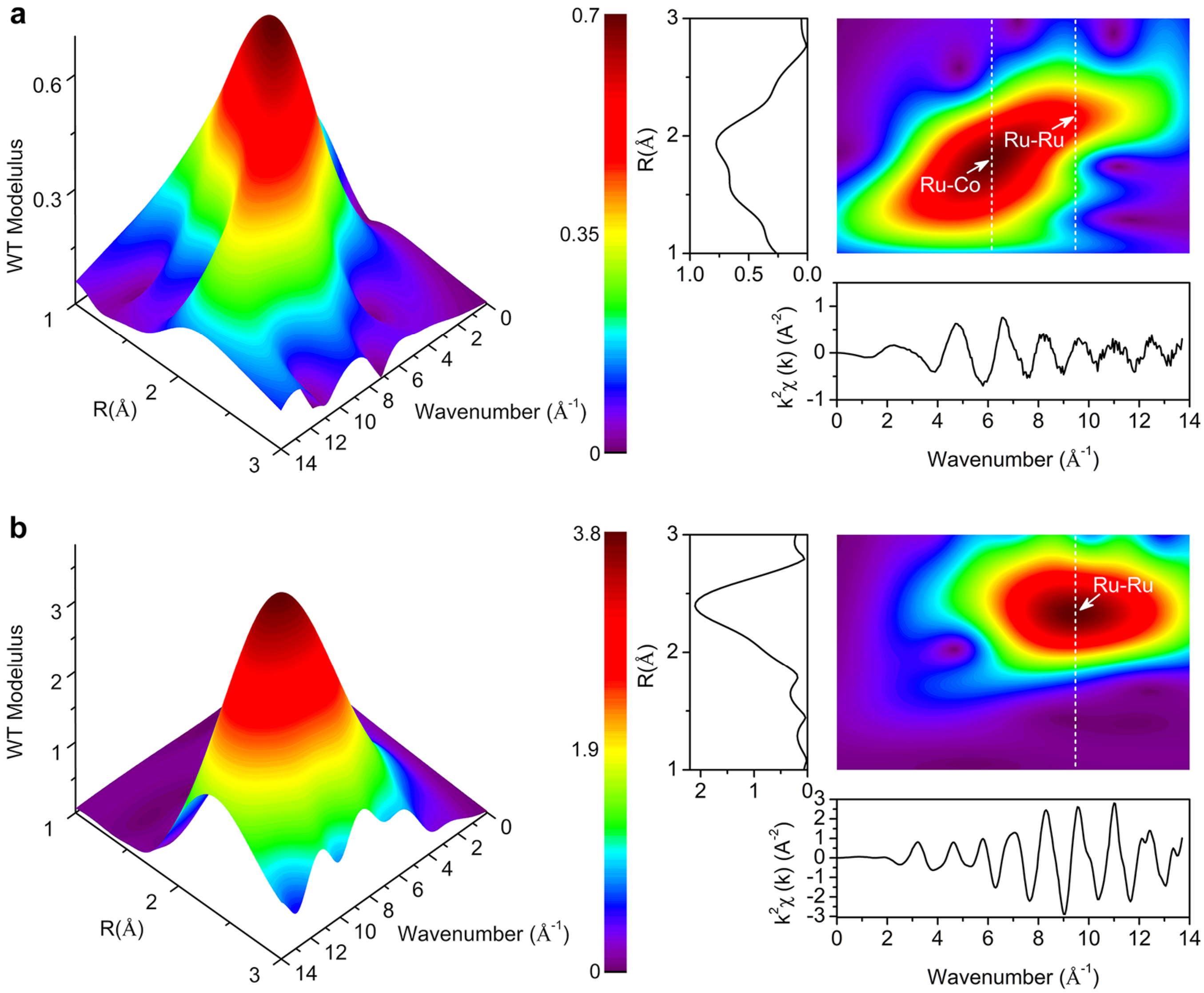

Supplementary Figure 9. (a) Wavelet transform (WT) for the k^2-weighted EXAFS signals for the Ru_nCo-NA catalyst; (b) Wavelet transform (WT) for the k^2-weighted EXAFS signals for Ru foil.

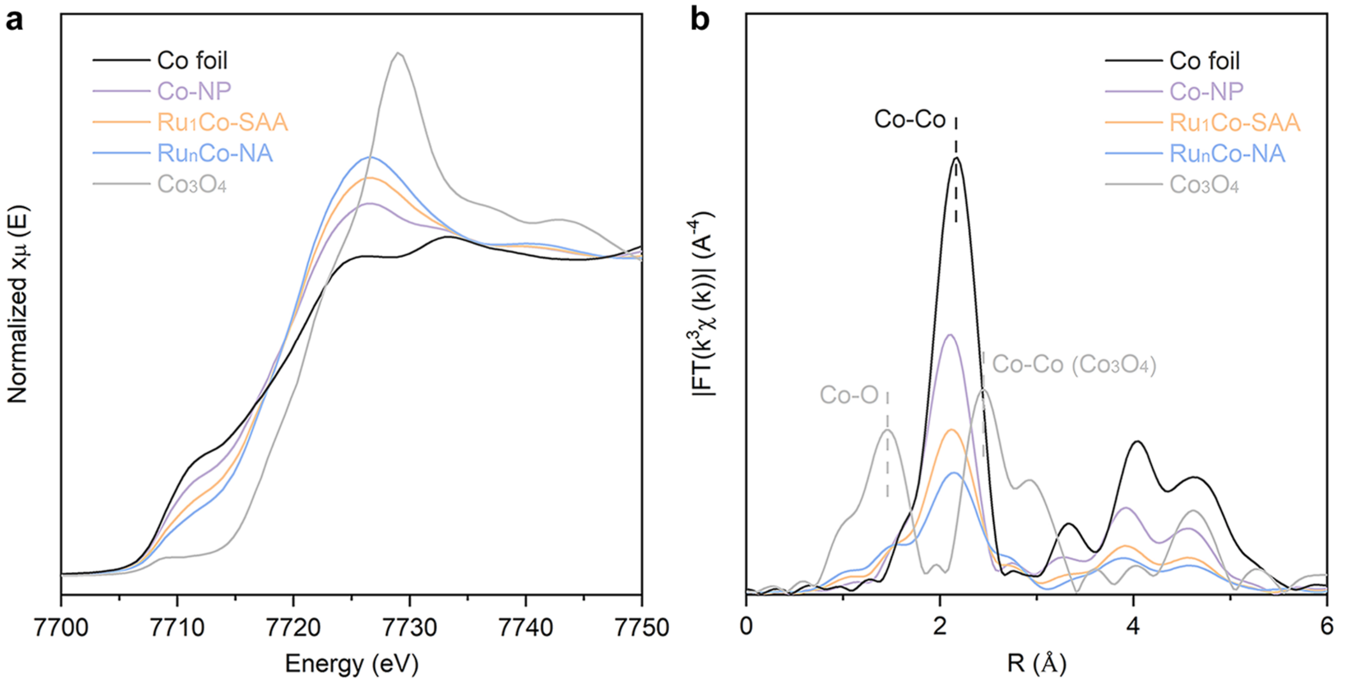

Supplementary Figure 10. (a) Co K-edge XANES profiles and (b) Co K-edge EXAFS spectra in *R* space for Co foil, Co-NP, Ru_1Co-SAA, Ru_nCo-NA and Co_3O_4.

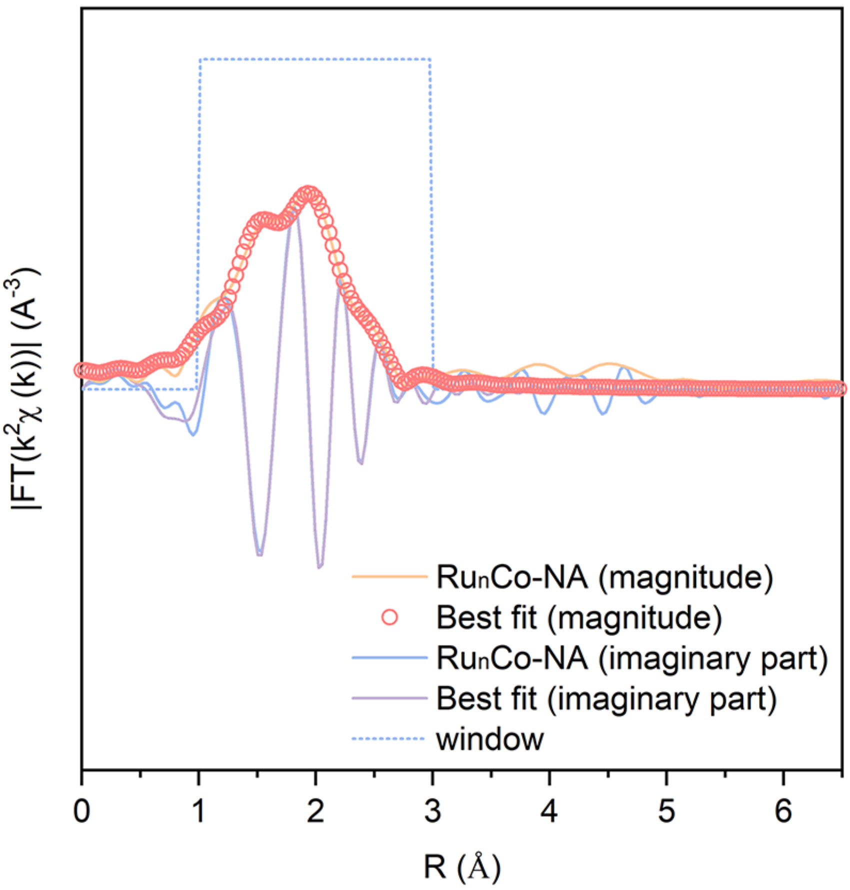

Supplementary Figure 11. Ru K-edge EXAFS fitting curves in *R* space for Ru_nCo-NA.

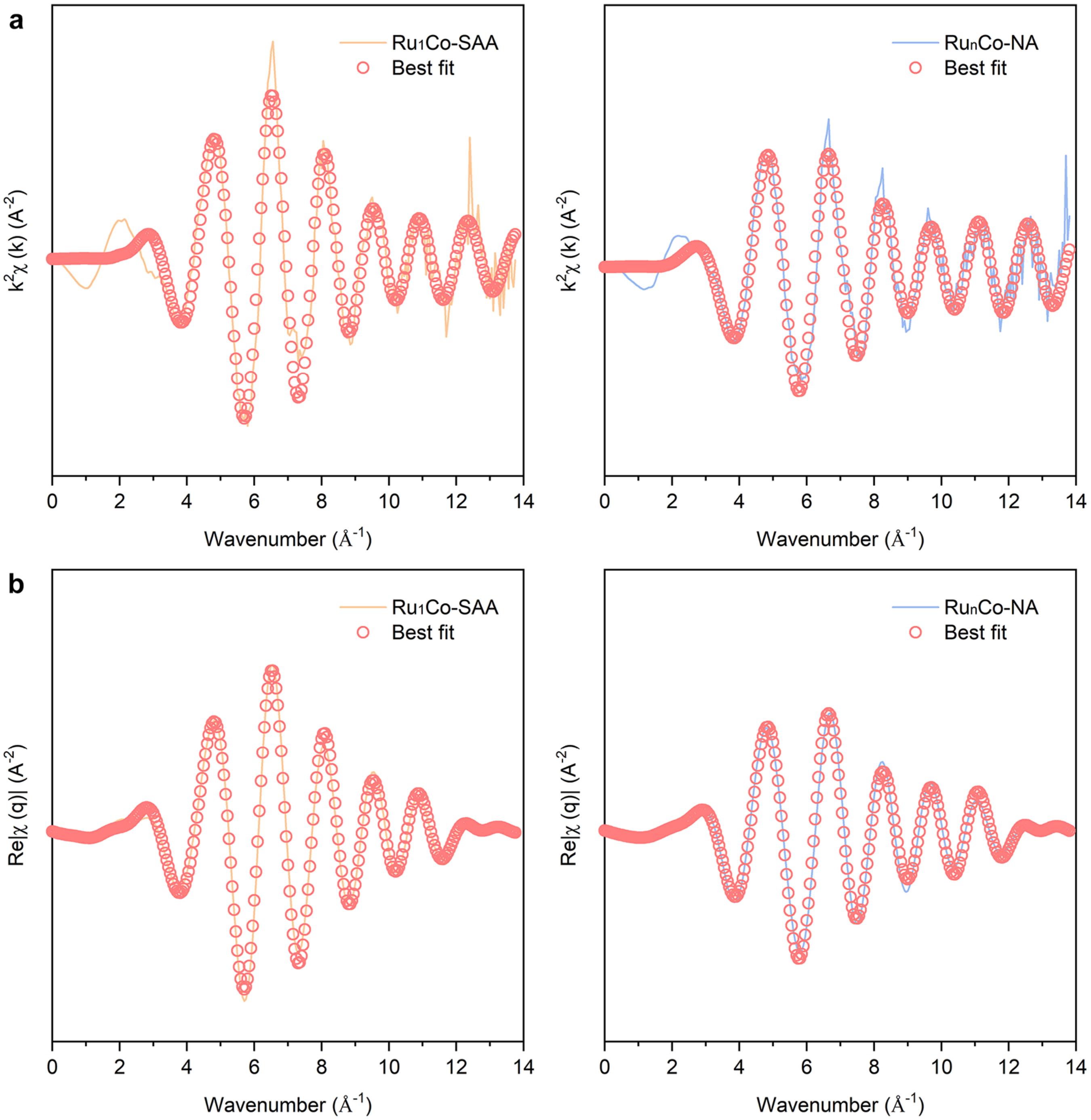

Supplementary Figure 12. Ru K-edge EXAFS fitting curves in (a) *k* space and (b) *q* space for the Ru_1Co-SAA and Ru_nCo-NA catalysts.

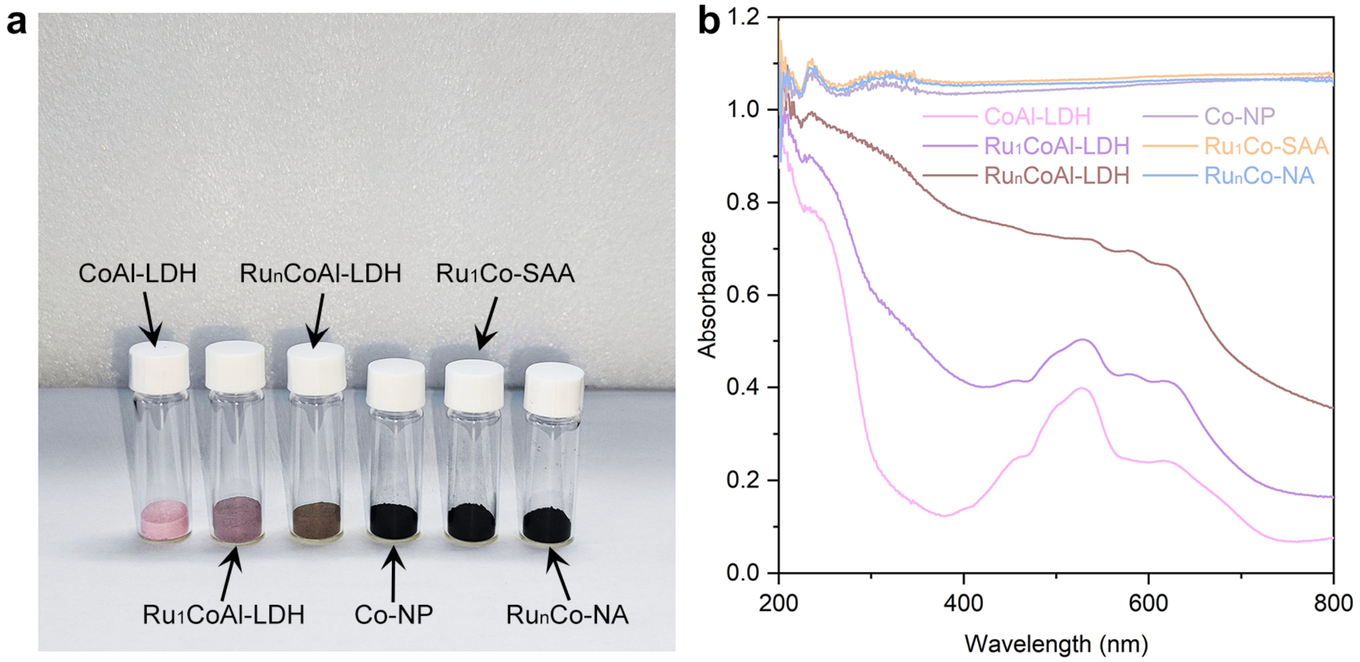

Supplementary Figure 13. (a) Digital paragraphs and (b) UV-Vis diffuse reflectance absorption spectra for the LDH precursors and the catalysts obtained by H_2-reduction of the LDH precursors at 650 °C.

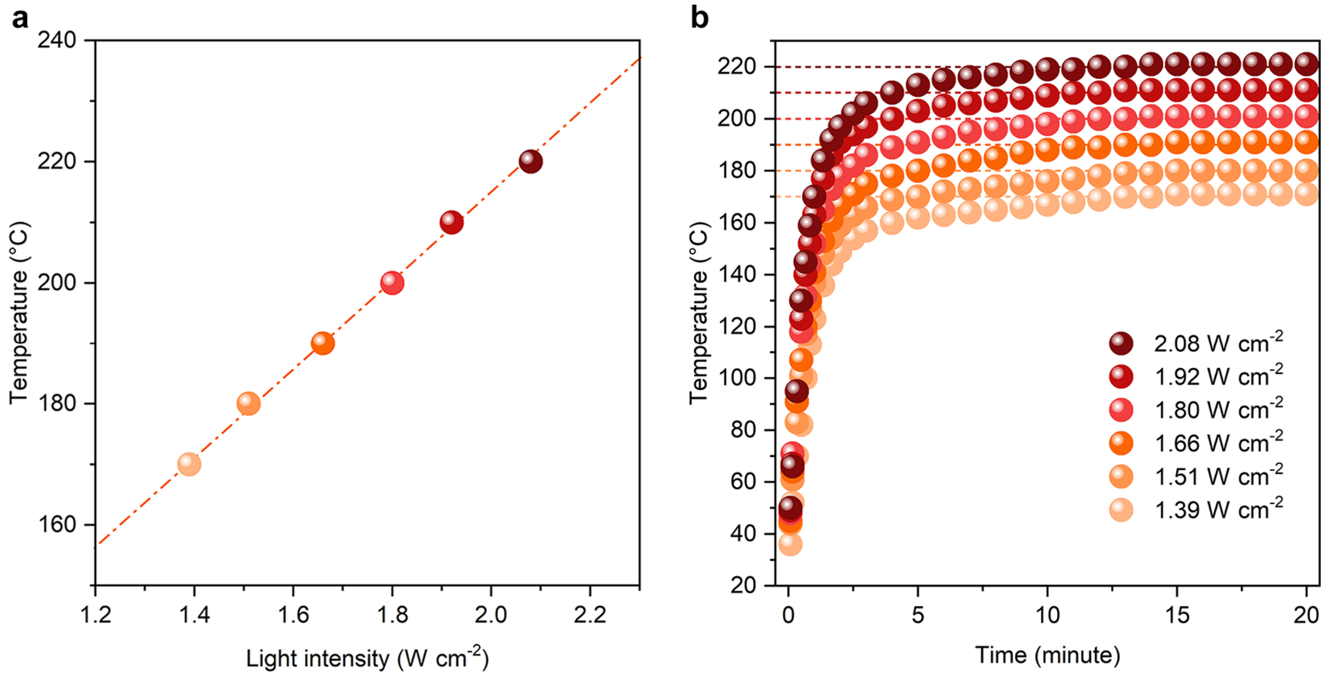

Supplementary Figure 14. Temperature profiles for CO photo-hydrogenation over the Ru_1Co-SAA catalyst under UV-Vis irradiation: (a) catalyst surface temperatures after 15 min under Xe lamp irradiation of different light intensity and (b) corresponding temperature rise curves.

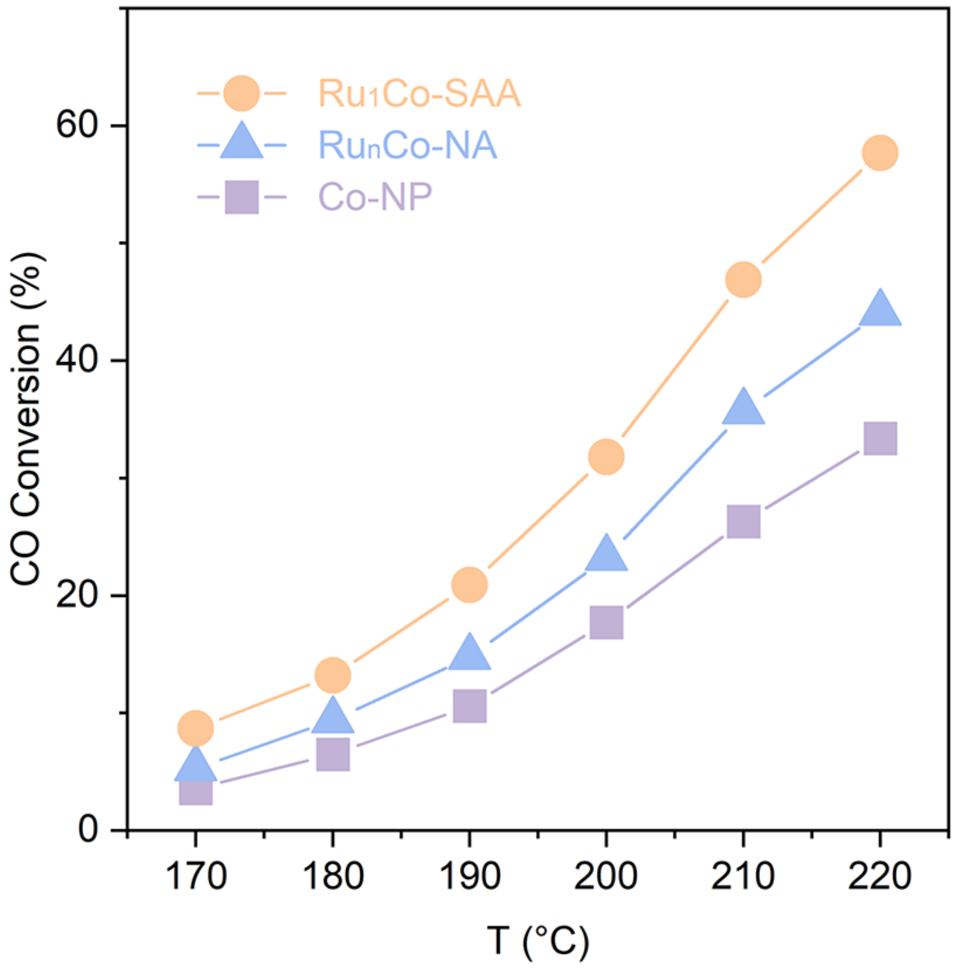

Supplementary Figure 15. CO conversion profile for CO photo-hydrogenation with Co-NP, Ru_1Co-SAA and Ru_nCo-NA catalysts at temperatures between 170-220 °C (reaction conditions: 50 mg catalyst, UV-Vis irradiation, light intensity from 1.39 W cm^{-2} (at 170 °C) to 2.08 W cm^{-2} (at 220 °C), 0.1 MPa, $CO/H_2/N_2$ = 20/40/40, GHSV = 2400 mL g^{-1} h^{-1}).

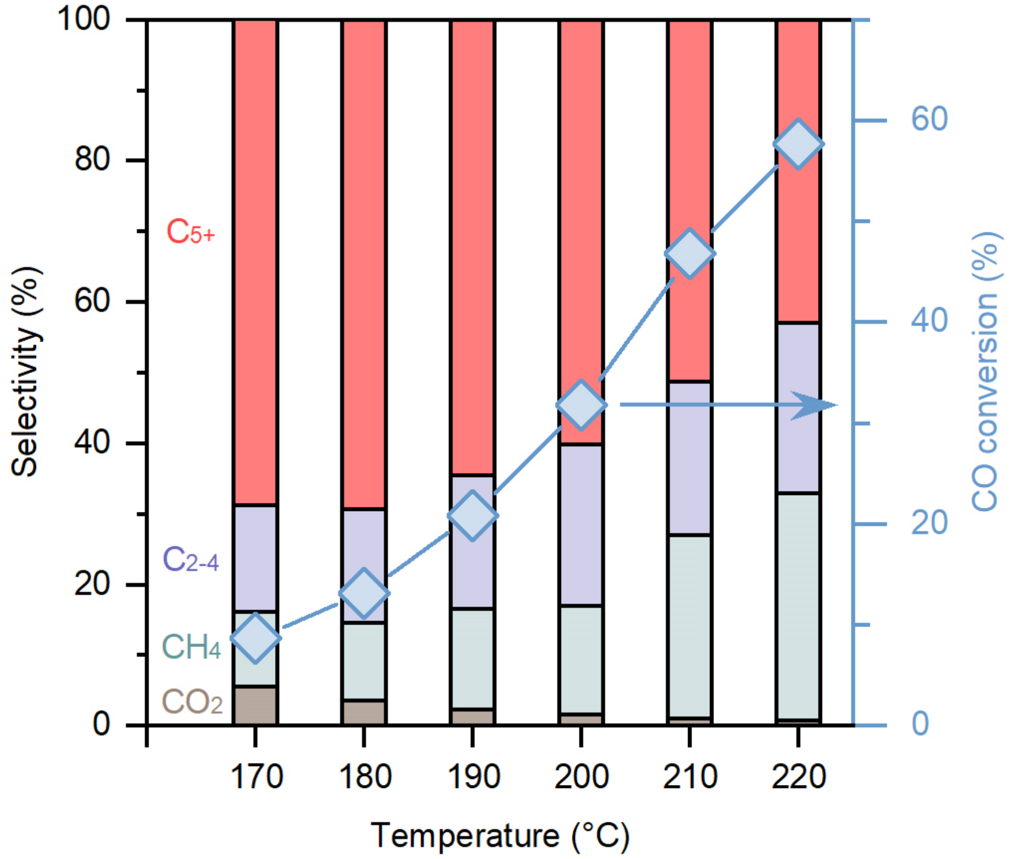

Supplementary Figure 16. Temperature-dependent product selectivity and CO conversion during CO photo-hydrogenation over Ru_1Co-SAA (reaction conditions: 50 mg catalyst, UV-Vis irradiation, light intensity from 1.39 W cm^{-2} (at 170 °C) to 2.08 W cm^{-2} (at 220 °C), 0.1 MPa, $CO/H_2/N_2$ = 20/40/40, GHSV = 2400 mL g^{-1} h^{-1}).

At UV-Vis intensities below 1.80 W cm^{-2}, the catalyst temperature remained below 200 °C, resulting in a low CO conversion (8.7% at 170 °C, 20.9% at 190 °C). At 1.80 W cm^{-2}, the temperature was 200 °C and the CO conversion was 31.8%. Further, the selectivity to valuable C_{2+} products relative to CO_2 at 200 °C was significantly higher than at temperatures below 200 °C. When the light intensity increased above 1.80 W cm^{-2}, the catalyst temperature increased which promoted methanation, leading to much higher selectivity towards CH_4 (26.0% at 210 °C and 32.1% at 220 °C). CO_2 and CH_4 are undesirable products in the FTS reactions due to their low value and greenhouse effects. Through comprehensive evaluation of CO conversion, C_{5+} selectivity, CH_4 selectivity and CO_2 selectivity, we identified 200 °C as the most appropriate temperature (achieved at a light intensity of 1.80 W cm^{-2} in our system) for photo-hydrogenating CO to C_{5+} liquid fuels.

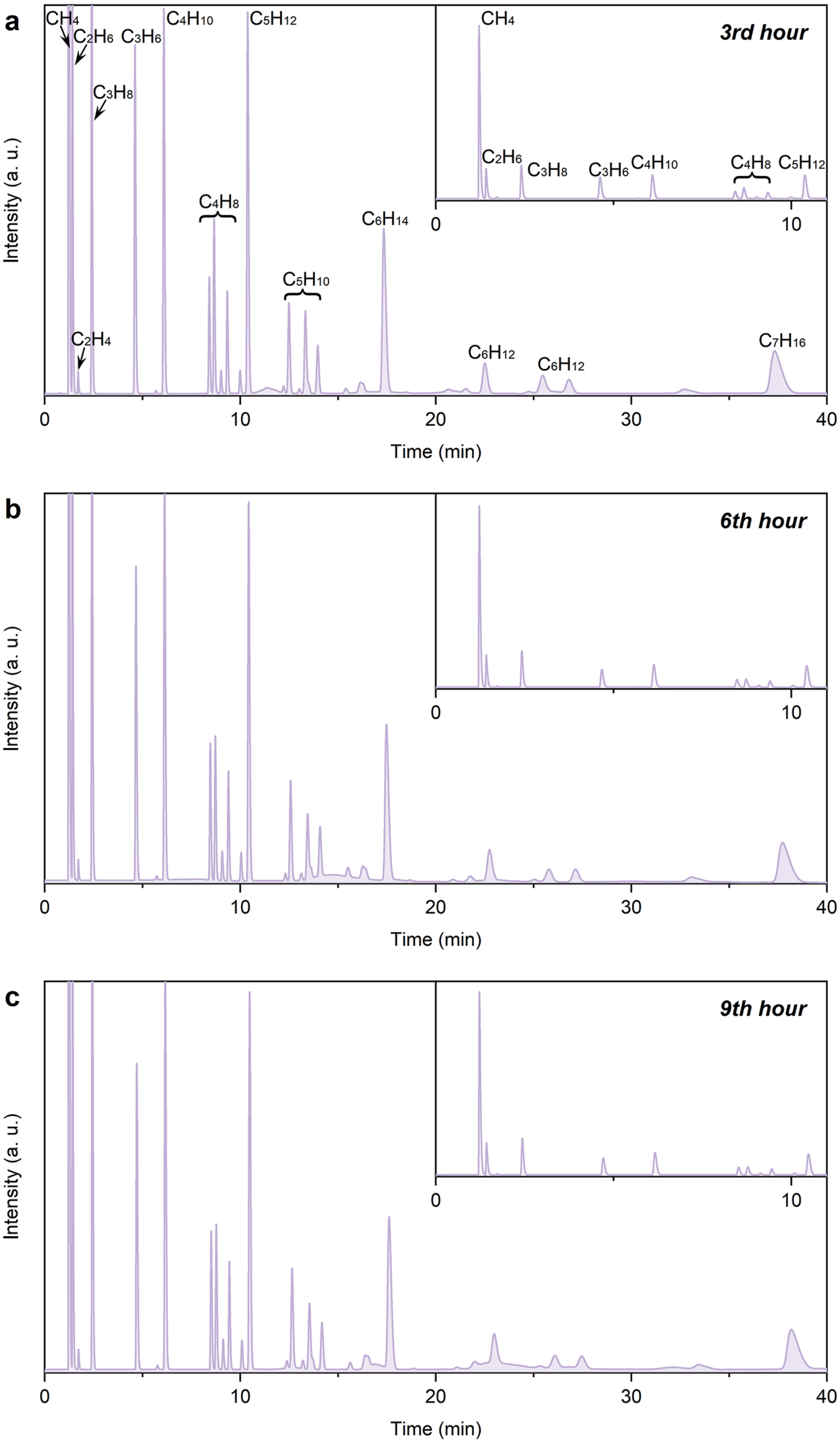

Supplementary Figure 17. GC spectrum of the gaseous products formed after (a) 3 h, (b) 6 h and (c) 9 h CO photo-hydrogenation using the Ru_1Co-SAA catalyst under 1.80 W cm^{-2} UV-Vis irradiation at 0.5 MPa.

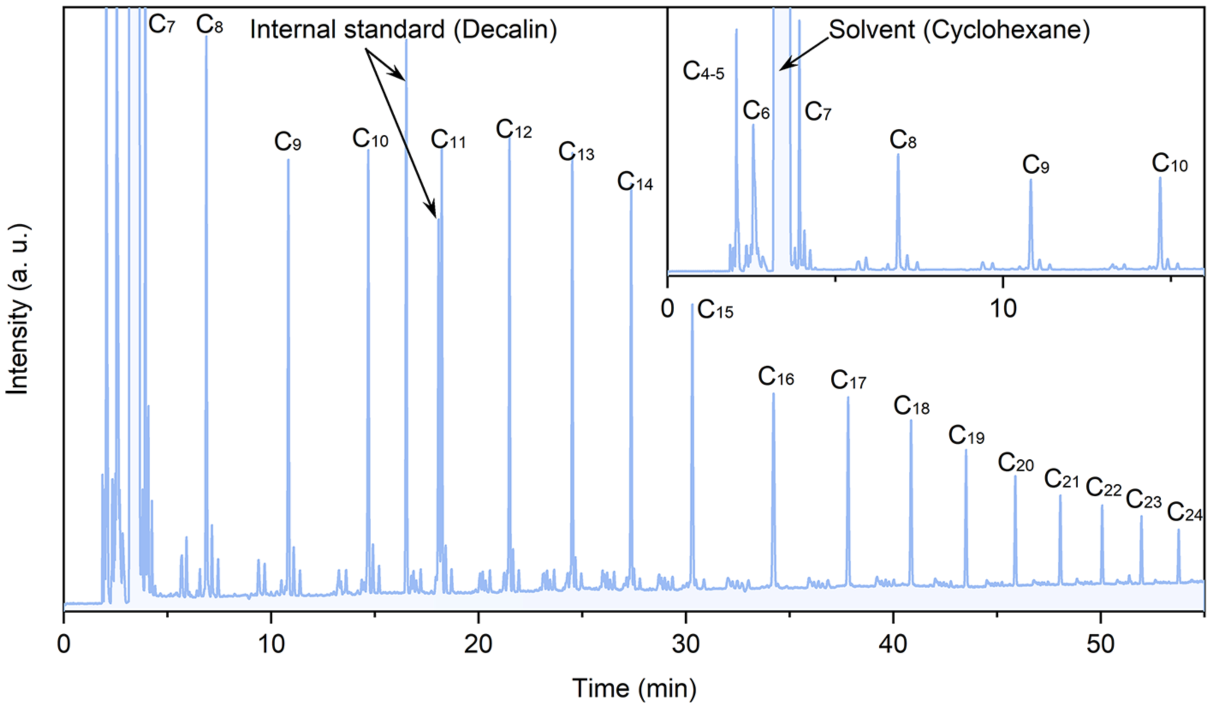

Supplementary Figure 18. GC spectrum of the liquid products collected in the cyclohexane phase after 10 h CO photo-hydrogenation with Ru_1Co-SAA under 1.80 W cm^{-2} UV-Vis irradiation at 0.5 MPa. The labels C_n indicate the carbon numbers in the alkane products.

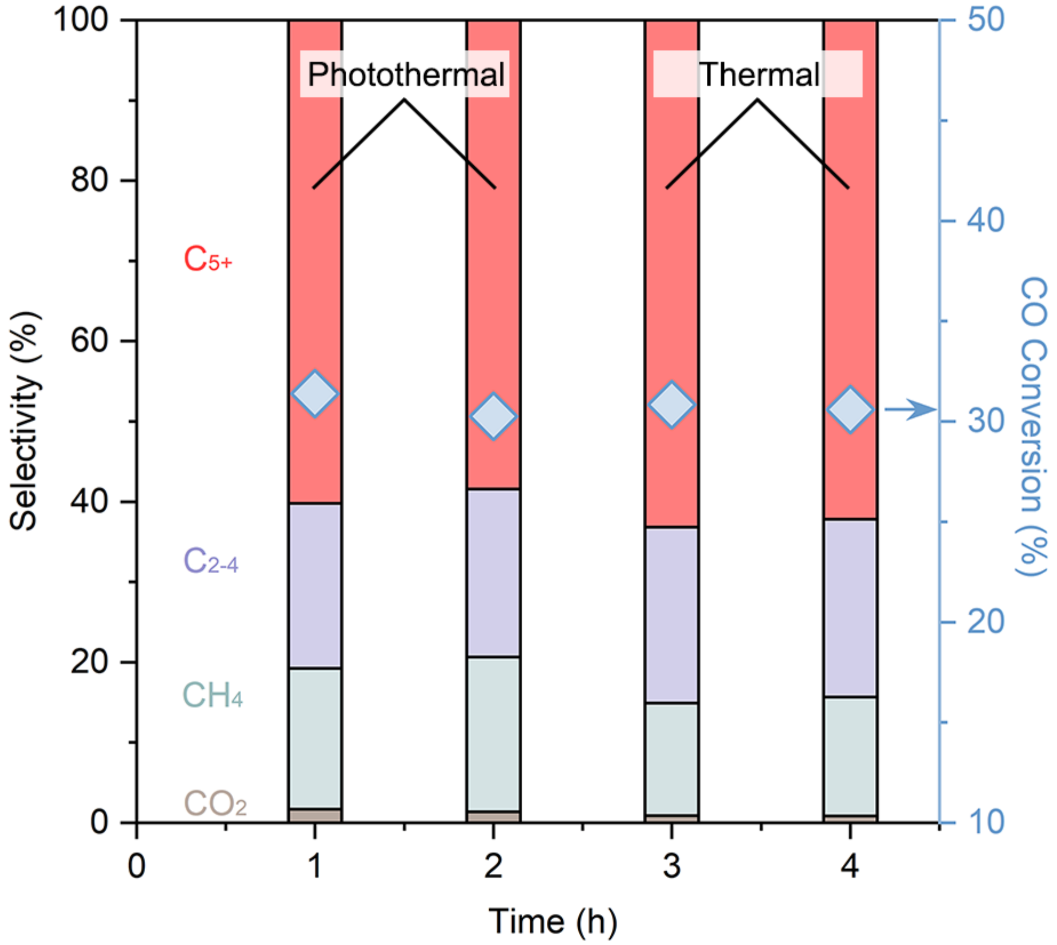

Supplementary Figure 19. Performance comparison of CO hydrogenation over Ru_1Co-SAA catalyst under UV-Vis irradiation and electric heating (dark) conditions.

In the continuous CO hydrogenation process, the catalyst surface was heated to 200 ^{o}C under 1.80 W cm^{-2} UV-Vis irradiation. After testing the performance of the Ru_1Co-SAA catalyst each hour for two hours, the Xe lamp light source was turned off and the catalyst cooled to room temperature. Then, without changing the flow rate or reloading the catalyst, the catalyst was electrically heated in the dark and the CO hydrogenation performance was evaluated over two hours. The CO conversion and product selectivity under direct electrical heating were almost identical to those determined under UV-Vis irradiation.

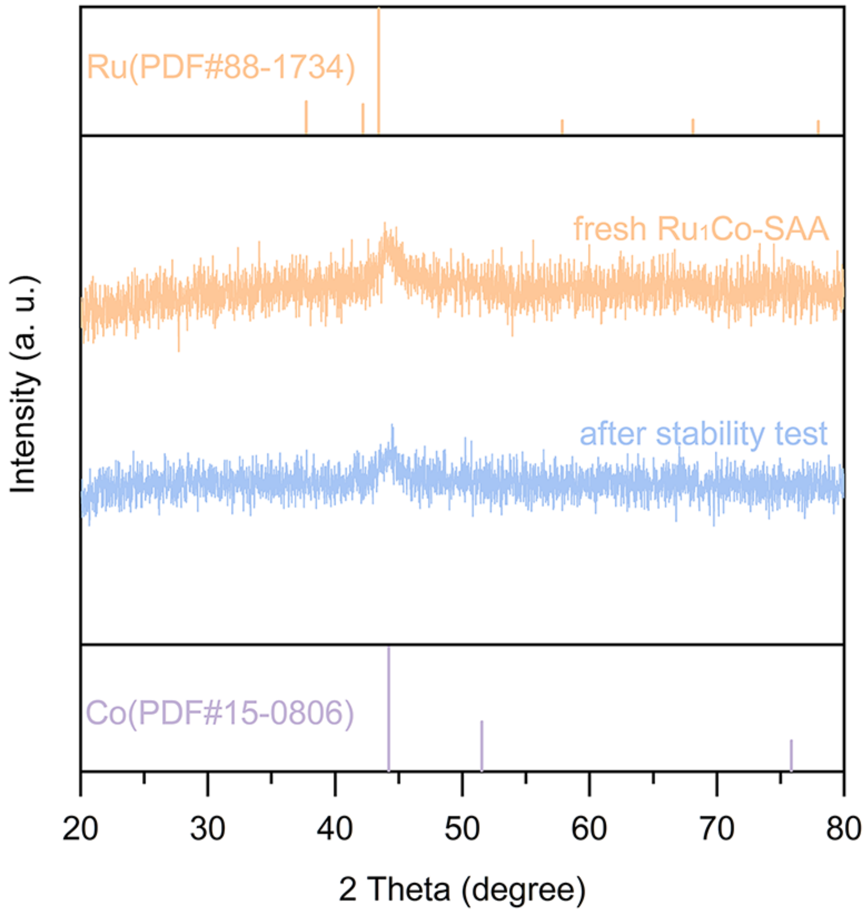

Supplementary Figure 20. Comparison of XRD patterns for the fresh Ru_1Co-SAA catalyst and the recycled Ru_1Co-SAA catalyst after the 100-hour stability test.

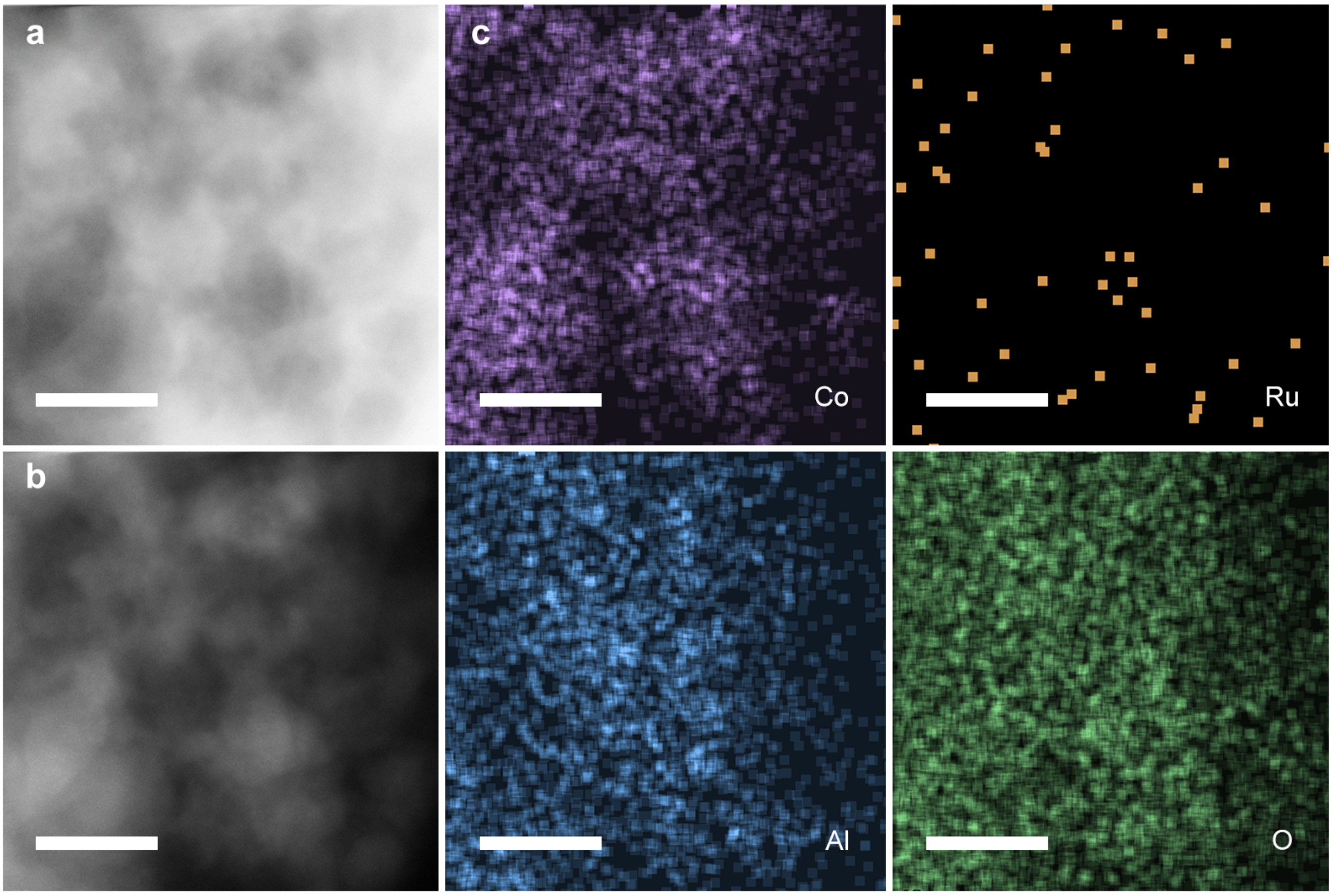

Supplementary Figure 21. HAADF-STEM images of recycled Ru_1Co-SAA after the 100-hour stability test: (a) ABF mode; (b) HAADF mode and (c) EDS element maps. Scale bar, 10 nm.

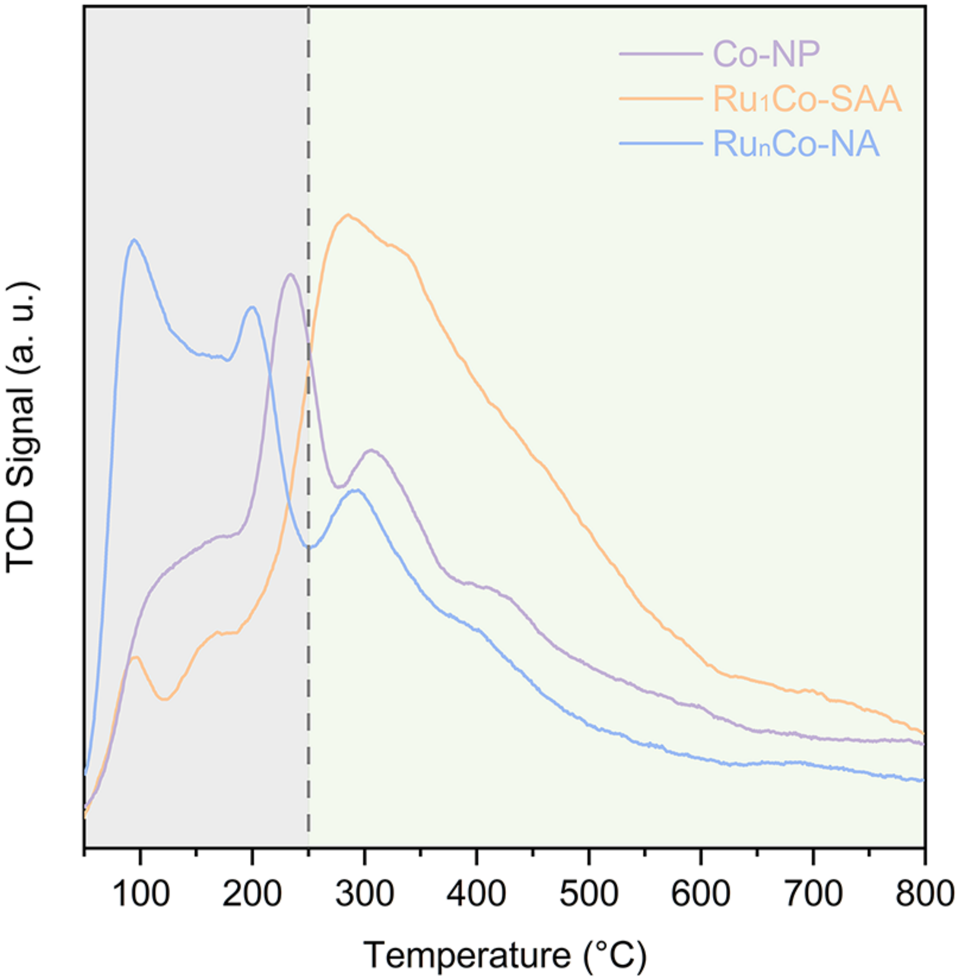

Supplementary Figure 22. CO-TPD profiles for the Co-NP, Ru_1Co-SAA and Ru_nCo-NA catalysts.

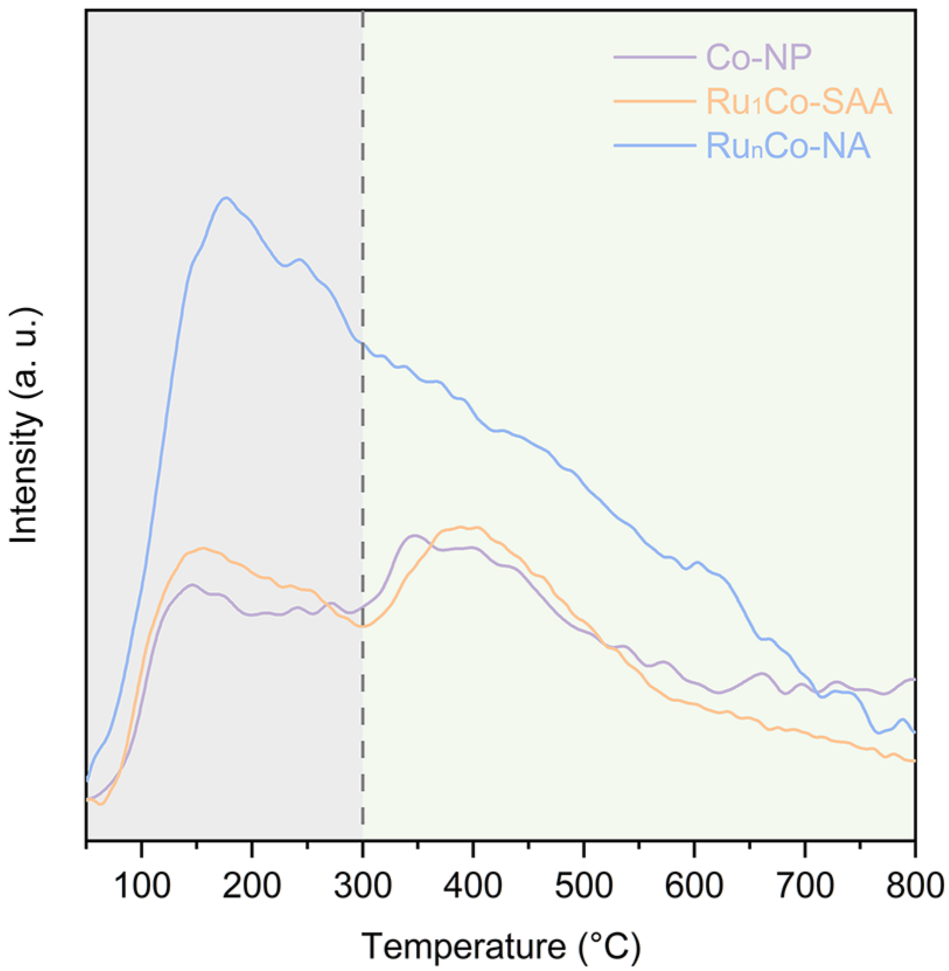

Supplementary Figure 23. H_2-TPD profiles for the Co-NP, Ru_1Co-SAA and Ru_nCo-NA catalysts.

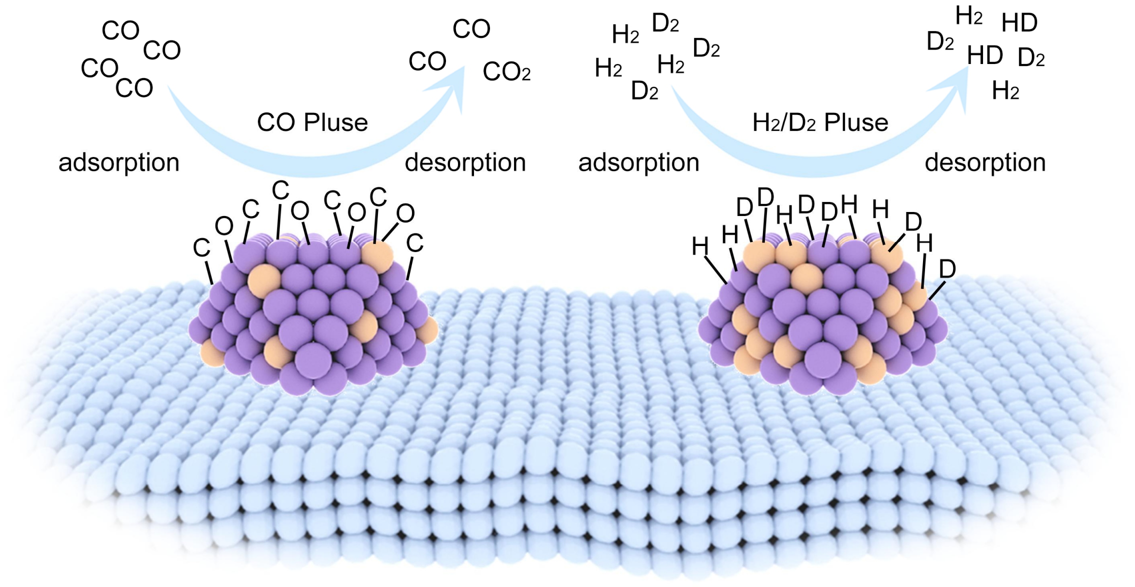

Supplementary Figure 24. Schematic mechanism of CO and H_2/D_2 pulse experiments.

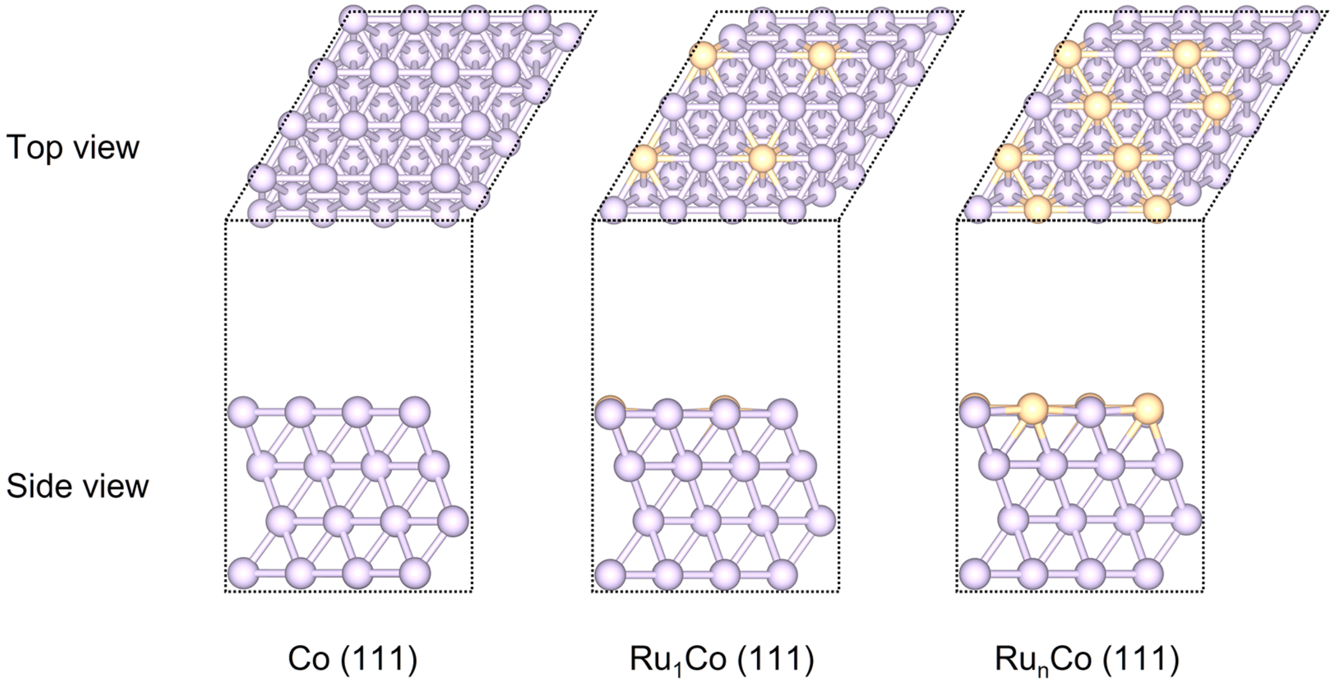

Supplementary Figure 25. The top and side views of the Co (111), Ru_1Co (111) and Ru_nCo (111) slab models. The violet and orange spheres represent Co and Ru atoms, respectively.

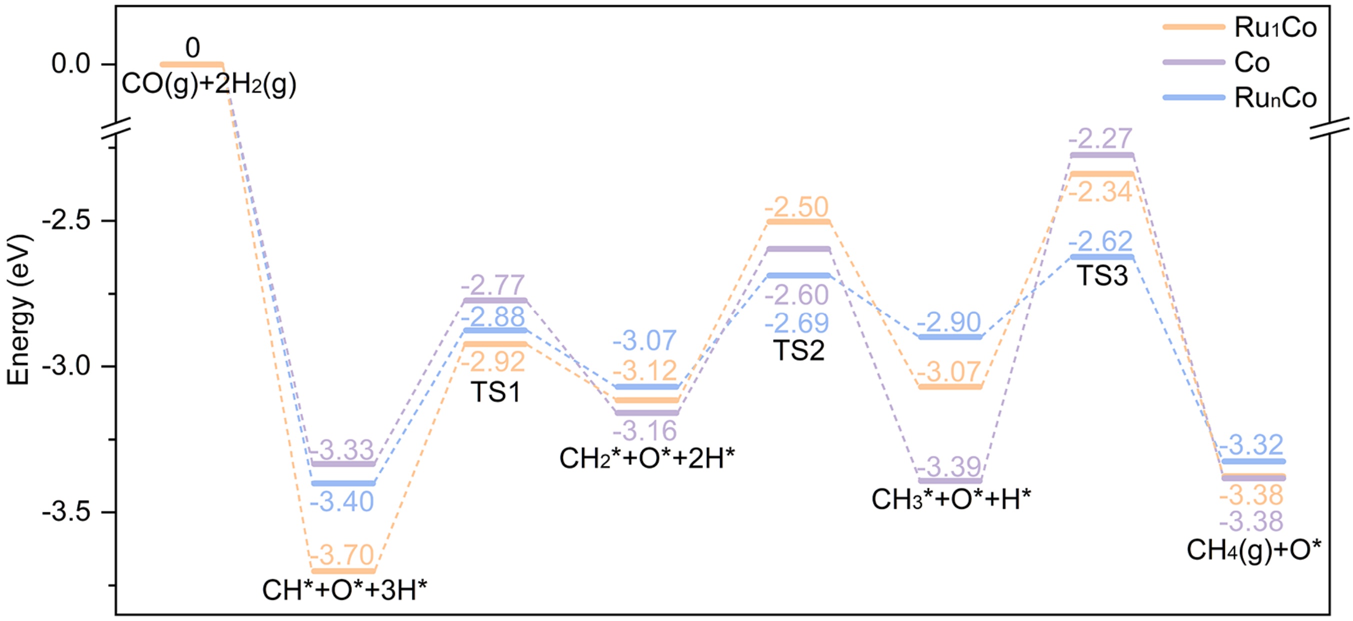

Supplementary Figure 26. Potential energy profile for CH* hydrogenation to CH_4.

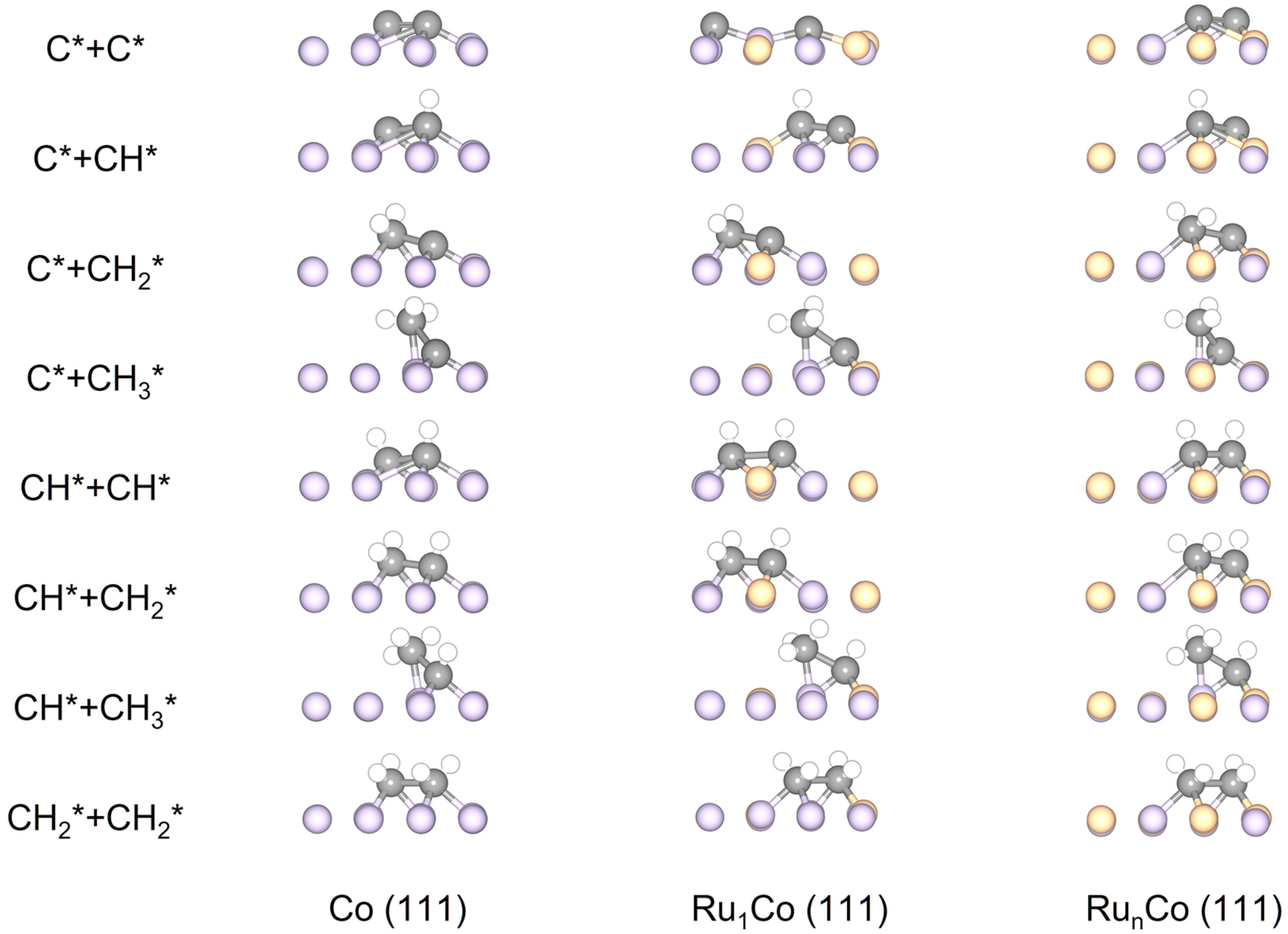

Supplementary Figure 27. The side views of transition state configurations for different C-C coupling paths. Color code: Ru (orange), Co (violet), C (grey), H (white).

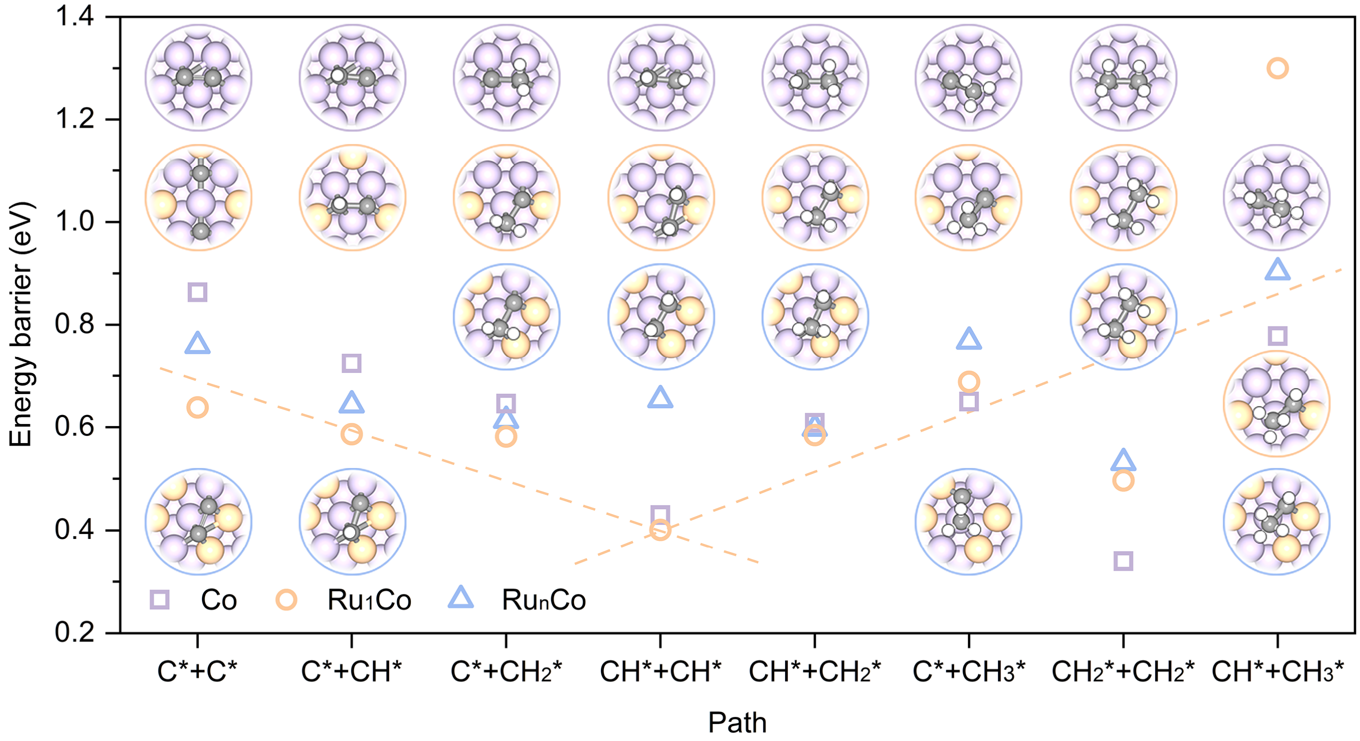

Supplementary Figure 28. Energy barriers of different C-C coupling paths with transition state configurations. Color code: Ru (orange), Co (violet), C (grey), H (white).

Supplementary Table 1. The mass ratio and molar ratio of Ru to Co in the LDH precursors and as-obtained catalysts, and metal contents in as-obtained catalysts, measured by ICP-OES.

Samples	Ru/Co mass ratio (wt.%)	Ru/Co molar ratio (%)	Co content (wt.%)	Ru content (wt.%)	Metal content (wt.%)
CoAl-LDH	0.00	0.00	30.95	0.00	/
Ru_1CoAl-LDH	2.65	1.53	26.35	0.70	/
Ru_nCoAl-LDH	11.34	6.61	27.55	3.12	/
Co-NP	0.00	0.00	51.61	0.00	51.61
Ru_1Co-SAA	2.87	1.66	51.92	1.49	53.41
Ru_nCo-NA	11.88	6.92	51.01	6.06	57.07

The molar ratio of Ru/Co in Ru_1CoAl-LDH determined by ICP-OES was 1.53:100. For Ru_nCoAl-LDH, the molar ratio was 6.61:100. After H_2 reduction, the Ru/Co ratios were very close (completely within the error range) to the LDH precursors, indicating the initial Ru/Co ratio was maintained during topological transformation of the LDHs to active catalysts.

Supplementary Table 2. BET surface area, BJH pore volumes and BJH pore diameters for different catalysts determined from N_2 adsorption measurements before CO hydrogenation tests.

Catalysts	S_{BET} (m^2 g^{-1})	V_{pore} (cm^3 g^{-1})	D_{pore} (nm)
Co-NP	147	0.40	3.42
Ru_1Co-SAA	149	0.42	3.80
Ru_nCo-NA	155	0.36	3.82

Supplementary Table 3. Ru K-edge EXAFS fitting results in *R* space for Ru foil, Ru_1Co-SAA and Ru_nCo-NA.

Samples	Paths	C.N.[a]	R (Å)[b]	σ^2 (10^{-3} Å^2)[c]	ΔE_0 (eV)[d]	R_{factor} (%)
Ru foil	Ru-Ru	12	2.68 ± 0.01	3.9	1.3	1.6
Ru_1Co-SAA	Ru-O	4.7 ± 0.4	2.07 ± 0.01	3.1	6.8	1.1
	Ru-Co	8.9 ± 1.3	2.53 ± 0.01	5.8	-9.2	
	Ru-Ru	N.A.	/	/	/	
Ru_nCo-NA	Ru-O	4.1 ± 1.2	2.01 ± 0.04	7.6	1.5	0.8
	Ru-Co	5.4 ± 0.9	2.54 ± 0.01	8.3	-5.3	
	Ru-Ru	2.6 ± 0.9	2.65 ± 0.04	7.1	-0.4	

[a] The coordination number; [b] average distance between absorber and backscattered atoms; [c] Debye-Waller factor; [d] the inner potential correction.

Supplementary Table 4. Performance comparison of different catalysts for CO photo-hydrogenation under UV-Vis irradiation.

Catalyst[a]	CO conversion (%)	Product selectivity (%)					
		CH_4	C_{2-4}	C_{5-12}	C_{13-20}	C_{21}^{+}	CO_2
CoAl-LDH	/	/	/	/	/	/	/
Ru_1CoAl-LDH	/	/	/	/	/	/	/
Ru_nCoAl-LDH	/	/	/	/	/	/	/
Co-NP	17.7	36.4	32.3	24.6	3.9	1.0	1.7
Ru_nCo-NA	23.2	23.7	32.2	33.5	7.2	1.5	2.0
Ru_1Co-SAA	31.8	15.4	22.8	47.3	11.4	1.5	1.5
Ru_1Co-SAA[b]	42.8	15.0	18.3	48.1	15.9	2.0	0.7
Ru_1Co-SAA[c]	58.6	11.2	12.6	47.5	24.1	4.2	0.3

[a] Reaction conditions: no external heating, $CO/H_2/N_2$ (20/40/40) as feed gas, GHSV = 2400 mL g^{-1} h^{-1}, 0.1 MPa (atmospheric pressure), 50 mg of catalyst, 300 W Xe lamp (UV-Vis light, 200-800 nm) as irradiation source, 10 h of irradiation time, 1.80 W cm^{-2} of irradiation intensity; [b] under pressure at 0.3 MPa and the same other conditions; [c] under pressure at 0.5 MPa and the same other conditions.

Supplementary Table 5. Performance comparison of Ru_1Co-SAA and various recently reported Co-based, Ru-based and RuCo-alloyed catalysts for photothermal and thermal FTS.

Catalyst	H_2/CO ratio	Temp. (°C)	Reactor type	Pressure (MPa)	GHSV (mL g^{-1} h^{-1})	CO Con. (%)	TOF (s^{-1})	C_{5+} Sel. (%)	CO_2 Sel. (%)	α[a]	Ref.
					photothermal						
**Ru_1Co-SAA**	**2**	**200**	**flow**	**0.5**	**2400**	**58.6**	**0.114**	**75.8**	**0.35**	**0.81**	**this study**
Co/Al_2O_3	3	210	batch[b]	0.18	N. P.	35.4	N. P.	28.7	3.1	0.61	1
Co/TiO_2 nanotube	2	220 + UV light	flow	2	1400	63.9	N. P.	42.7	17.3	N. P.	2
Ru/Graphene	2	150	batch[c]	3	N. P.	43	N. P.	81.7	< 1	0.78	3
10 wt.% Ru/SiC	2	150	batch[c]	3	N. P.	N. P.	0.0023	68	6	N. P.	4
					thermal						
Co/Ymeso-Na						32		84			
Co/Ymicro-Na	1	250	flow	2	2160	36	N. P.	77.1	N. P.	N. P.	5
Co/Ymeso-Ce						34		82.6			
Co/SiO_2-8h						78.2	0.049	80		0.77	
Co/SiO_2-12h	2	220	flow	2	4392	80.6	0.064	74.9	N. P.	0.73	6
Co/SiO_2-IM						84	0.065	67.3		N. P.	
CoRu0.2/Ti	2	220	flow	2	N. P.	10	0.093	56.8	N. P.	0.78	7
CoRu/MHCS	2	220	flow	1	1800	19.4	0.0036	63.7	N. P.	0.76	8
Co/MHCS						11.2	0.0014	72		0.81	
Co/Al_2O_3	2	220	flow	2	1249	12.5	0.02	85.8	N. P.	N. P.	9
Co/C	2	235	flow	1	5550	57	0.04	58	N. P.	0.68	10

[a] ASF chain growth probability; [b] gas-phase batch-type reaction; [c] aqueous-phase batch-type reaction.

Supplementary Table 6. Energy barriers (E_a, eV) and reaction energies (E_r, eV) for CH_4 formation on the Co (111) surface.

Paths	E_a (eV)	E_r (eV)
$CO^*+H^* \rightarrow HCO^*$	1.32	1.05
$HCO^* \rightarrow CH^*+O^*$	0.81	-0.71
$CH^*+H^* \rightarrow CH_2^*$	0.56	0.17
$CH_2^*+H^* \rightarrow CH_3^*$	0.56	-0.23
$CH_3^*+H^* \rightarrow CH_4(g)$	1.12	0.01

Supplementary Table 7. Energy barriers (E_a, eV) and reaction energies (E_r, eV) for CH_4 formation on the Ru_1Co (111) surface.

Paths	E_a (eV)	E_r (eV)
$CO^*+H^* \rightarrow HCO^*$	1.17	0.97
$HCO^* \rightarrow CH^*+O^*$	0.65	-0.69
$CH^*+H^* \rightarrow CH_2^*$	0.78	0.58
$CH_2^*+H^* \rightarrow CH_3^*$	0.61	0.05
$CH_3^*+H^* \rightarrow CH_4(g)$	0.73	-0.31

Supplementary Table 8. Energy barriers (E_a, eV) and reaction energies (E_r, eV) for CH_4 formation on the Ru_nCo (111) surface.

Paths	E_a (eV)	E_r (eV)
$CO^*+H^* \rightarrow HCO^*$	1.39	1.29
$HCO^* \rightarrow CH^*+O^*$	0.55	-0.84
$CH^*+H^* \rightarrow CH_2^*$	0.52	0.33
$CH_2^*+H^* \rightarrow CH_3^*$	0.38	0.17
$CH_3^*+H^* \rightarrow CH_4(g)$	0.28	-0.43

Supplementary Table 9. Energy barriers (E_a, eV) and reaction energies (E_r, eV) for CH_x* coupling on the Co (111) surface.

Paths	E_a (eV)	E_r (eV)
C*+C*→CC*	0.86	-0.58
C*+CH*→CCH*	0.72	-0.67
C*+CH_2*→CCH_2*	0.65	-0.90
C*+CH_3*→CCH_3*	0.65	-1.19
CH*+CH*→CHCH*	0.43	0.32
CH*+CH_2*→$CHCH_2$*	0.61	-0.21
CH*+CH_3*→$CHCH_3$*	0.78	-0.28
CH_2*+CH_2*→CH_2CH_2*	0.34	-0.66

Supplementary Table 10. Energy barriers (E_a, eV) and reaction energies (E_r, eV) for CH_x* coupling on the Ru_1Co (111) surface.

Paths	E_a (eV)	E_r (eV)
C*+C*→CC*	0.64	-0.30
C*+CH*→CCH*	0.59	-0.35
C*+CH_2*→CCH_2*	0.58	-1.02
C*+CH_3*→CCH_3*	0.69	-1.43
CH*+CH*→CHCH*	0.40	-0.53
CH*+CH_2*→$CHCH_2$*	0.58	-0.12
CH*+CH_3*→$CHCH_3$*	1.30	0.14
CH_2*+CH_2*→CH_2CH_2*	0.50	-0.42

Supplementary Table 11. Energy barriers (E_a, eV) and reaction energies (E_r, eV) for CH_x* coupling on the Ru_nCo (111) surface.

Paths	E_a (eV)	E_r (eV)
C*+C*→CC*	0.76	-0.92
C*+CH*→CCH*	0.64	-0.53
C*+CH_2*→CCH_2*	0.61	-1.08
C*+CH_3*→CCH_3*	0.77	-1.25
CH*+CH*→CHCH*	0.65	-0.16
CH*+CH_2*→$CHCH_2$*	0.60	-0.17
CH*+CH_3*→$CHCH_3$*	0.90	-0.22
CH_2*+CH_2*→CH_2CH_2*	0.53	-0.48

Supplementary Table 12. Results of correlation analysis including Pearson correlation coefficients (σ) and p-values based on E_a, E_r, Ω and Ru/Co ratio on the catalytic surface.

Factors	Pearson correlations	ΔE_a	ΔE_r	Ω	Ru/Co ratio
ΔE_a	σ	1	0.16	-0.75	-0.069
	p-value	/	0.55	8.1E-4	0.80
ΔE_r	σ	0.16	1	-0.21	0.16
	p-value	0.55	/	0.43	0.57
Ω	σ	-0.75	-0.21	1	-1.7E-16
	p-value	8.1E-4	0.43	/	1
Ru/Co ratio	σ	-0.069	0.16	-1.7E-16	1
	p-value	0.80	0.56646	1	/

Supplementary References

1 Li, Z. et al. Photothermal hydrocarbon synthesis using alumina-supported cobalt metal nanoparticle catalysts derived from layered-double-hydroxide nanosheets. *Nano Energy* **60**, 467-475 (2019).

2 Wang, L., Zhang, Y., Gu, X., Zhang, Y. & Su, H. Insight into the role of UV-irradiation in photothermal catalytic Fischer-Tropsch synthesis over TiO_2 nanotube-supported cobalt nanoparticles. *Catal. Sci. Technol.* **8**, 601-610 (2018).

3 Guo, X.-N., Jiao, Z.-F., Jin, G.-Q. & Guo, X.-Y. Photocatalytic Fischer-Tropsch synthesis on graphene-supported worm-like ruthenium nanostructures. *ACS Catal.* **5**, 3836-3840 (2015).

4 Liang, Z.-P. et al. Aldehydes rather than alcohols in oxygenated products from light-driven Fischer-Tropsch synthesis over Ru/SiC catalysts. *Catal. Sci. Technol.* **9**, 4629-4632 (2019).

5 Li, J. et al. Integrated tuneable synthesis of liquid fuels via Fischer-Tropsch technology. *Nat. Catal.* **1**, 787-793 (2018).

6 Cheng, Q. et al. Confined small-sized cobalt catalysts stimulate carbon-chain growth reversely by modifying ASF law of Fischer-Tropsch synthesis. *Nat. Commun.* **9**, 3250 (2018).

7 Bertella, F. et al. Insights into the promotion with Ru of Co/TiO_2 Fischer-Tropsch catalysts: an in situ spectroscopic study. *ACS Catal.* **10**, 6042-6057 (2020).

8 Phaahlamohlaka, T. N. et al. Effects of Co and Ru intimacy in Fischer-Tropsch catalysts using hollow carbon sphere supports: assessment of the hydrogen spillover processes. *ACS Catal.* **7**, 1568-1578 (2017).

9 Hernández Mejía, C., van Deelen, T. W. & de Jong, K. P. Activity enhancement of cobalt catalysts by tuning metal-support interactions. *Nat. Commun.* **9**, 4459 (2018).

10 Luo, Q.-X. et al. Cobalt nanoparticles confined in carbon matrix for probing the size dependence in Fischer-Tropsch synthesis. *J. Catal.* **369**, 143-156 (2019).
